# Deciphering the Energy Transfer Mechanism Across Metal Halide Perovskite‐Phthalocyanine Interfaces

**DOI:** 10.1002/advs.202414831

**Published:** 2025-01-10

**Authors:** Alejandro Cortés‐Villena, Alejandro Cadranel, Kobra Azizi, Tomás Torres, Dirk M. Guldi, Julia Pérez‐Prieto, Raquel E. Galian

**Affiliations:** ^1^ Institute of Molecular Science University of Valencia c/Catedrático José Beltrán Martínez 2 Paterna 46980 Valencia Spain; ^2^ FAU Profile Center SolarDepartment of Chemistry and Pharmacy & Interdisciplinary Center for Molecular Materials (ICMM) Friedrich‐Alexander‐Universität Erlangen‐Nürnberg (FAU) Egerlandstrasse 3 91058 Erlangen Germany; ^3^ CONICET – Instituto de Química Física de Materiales Medio Ambiente y Energía (IN‐QUIMAE) Universidad de Buenos Aires Viamonte 430 Buenos Aires 1053 Argentina; ^4^ Facultad de Ciencias Exactas y Naturales Departamento de Química Inorgánica Analítica y Química Física Universidad de Buenos Aires Viamonte 430 Buenos Aires 1053 Argentina; ^5^ Blue World Technologies Egeskovvej 6C Kvistgaard 3490 Denmark; ^6^ Department of Organic Chemistry and Institute for Advanced Research in Chemical Sciences (IAdChem) Universidad Autónoma de Madrid Cantoblanco Madrid 28049 Spain; ^7^ IMDEA‐Nanociencia Universidad Autónoma de Madrid c/Faraday, 9, Cantoblanco Madrid 28049 Spain; ^8^ Department of Organic Chemistry University of Valencia Av. Vicent Andrés Estellés s/n Burjassot 46100 Valencia Spain

**Keywords:** perovskite nanocrystals, perovskite‐phthalocyanine nanohybrids, singlet energy transfers, time‐resolved photoluminescence, transient absorption spectroscopy

## Abstract

Energy transfer processes in nanohybrids are at the focal point of conceptualizing, designing, and realizing novel energy‐harvesting systems featuring nanocrystals that absorb photons and transfer their energy unidirectionally to surface‐immobilized functional dyes. Importantly, the functionality of these dyes defines the ultimate application. Herein, CsPbBr_3_ perovskite nanocrystals (NCs) are interfaced with zinc phthalocyanine (ZnPc) dyes featuring carboxylic acid. The functionality is the photosensitization of singlet oxygen. The CsPbBr_3_@ZnPc nanohybrid is to the best of our knowledge the first example, in which an unusual Dexter‐type singlet energy transfer between metal halide perovskite nanocrystals and phthalocyanine dyes enables singlet oxygen generation as a proof‐of‐concept application. A detailed temporal picture of the singlet energy transfer mechanism is made possible by combining key time‐resolved spectroscopic techniques, that are, femtosecond, nanosecond, and microsecond transient absorption spectroscopy as well as time‐correlated single photon counting, and target analyses. In fact, three excitonic components in the NCs govern a concerted Dexter‐type energy transfer. The work illustrates the potential of CsPbBr_3_@ZnPc as a singlet photosensitizer of ZnPc to produce singlet oxygen (^1^O_2_) almost quantitatively while photoexciting CsPbBr_3_.

## Graphical abstract

This study presents photophysical insights into the energy transfer mechanism between CsPbBr_3_ perovskite nanocrystals (NCs) interfaced with zinc phthalocyanine (ZnPc) dyes by an unusual Dexter‐type singlet energy transfer, generating singlet oxygen as a proof‐of‐concept, using an arsenal of key time‐resolved spectroscopic techniques, such as femtosecond, nanosecond, and microsecond transient absorption spectroscopies as well as time‐correlated single photon counting, and target analyses.

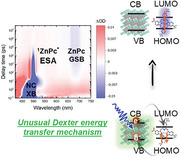

## 1 Introduction

The design and development of materials that mimic biological systems, by which they efficiently harvest, convert, and store visible photons is a contemporary challenge.^[^ ^1^ ^]^ To this end, a diverse platform of nanomaterials including semiconductor quantum dots, metal nanoparticles, and organic‐inorganic heterostructures among others, has emerged. Chalcogenide semiconductor quantum dots (QDs) are efficient antenna materials, whose optical and electronic properties depend on size, shape, and composition. Metal halide perovskite nanocrystals (NC) outperform chalcogenide QDs. What stands out are their superior absorption coefficients, higher photoluminescence quantum yields, tunable conduction and valence bands through halide alloying, and defect tolerance.^[^ ^2^ ^]^

Integrating semiconductor nanocrystals together with organics that are photo‐ and redox‐active has been at the forefront in photovoltaic, light‐emitting devices, and sensing applications. Decisive is an in‐depth understanding of photoinduced processes such as exciton dynamics, charge transfer, and energy transfer. This is crucial for the design of advanced materials with tailored properties for applications in the areas of photon upconversion and photocatalysis,^[^ ^3^ ^]^ among others. A common denominator thereof is the development of sustainable energy solutions.

Although perovskite nanocrystals have been used in the past as photosensitizers, few examples involve singlet energy transfer. Förster‐type singlet energy‐transfer mechanism is the most common mechanism that is operative in these nanohybrids.^[^ ^4^ ^]^ Here, the spectral overlap between the emission of the energy donor and the absorbance of the energy acceptor, accompanied by strong oscillator strengths for these transitions, is crucial. Leading examples include i) CsPbBr_3_‐rhodamine, CsPbBr_3_‐rhodamine isothiocyanate, CsPbBr_3_‐RoseBengal;^[^ ^5^ ^]^ ii) CsPbI_3_–cyanine dye;^[^ ^6^ ^]^ iii) CsPbBr_3_‐BODIPY;^[^ ^7^ ^]^ iv) CsPbBr_3_‐perylene diimide;^[^ ^8^ ^]^ v) CsPbX_3_‐anthracene (X: Br,I);^[^ ^9^ ^]^ and vi) CsPbBr_3_‐rubrene.^[^ ^10^ ^]^ However, far fewer reports deal with a Dexter‐type singlet energy transfer. Kamat et. al., for example, have suggested that this mechanism is active when a mixed halide perovskite CsPb(Br_1‐x_Cl_x_)_3_ as energy donor is combined with Rhodamine‐B as energy acceptor. A rationale is the poor spectral overlap (*J*).^[^ ^5a^ ^]^ Taking CsPbBr_3_‐pentacene for example, a sequential Dexter‐like electron exchange across the CsPbBr_3_/pentacene interface was proposed. An initial ultrafast hole transfer process is followed by a slow electron transfer.^[^ ^11^ ^]^ Recently, a Dexter exchange‐type interaction, with a minimal donor‐acceptor spectral overlap, was proposed for the attachment of dyes containing dimethyl iminium as an anchoring group. However, due to the lack of transient absorption experiments, this study was blind to dark states.^[^ ^12^ ^]^ Understanding the dynamics of energy transfer remains a key challenge, especially when considering that a singlet energy transfer based on perovskite nanocrystals triggers applications.

By virtue of a rich and tunable photo‐ as well as redox chemistry, phthalocyanine (Pc) dyes have attracted tremendous attention. It is their conjugated 18 π‐electron system that makes the difference.^[^ ^13^ ^]^ For example, Pcs exhibit molar absorption coefficients as high as 10^5^ m ^−1^ cm^−1^ in the 650–700 nm region of the solar spectrum next to thermal and chemical stability.^[^ ^14^ ^]^ These and other facts render Pcs efficient near‐infrared light converters. As a matter of fact, they have been used in electron donor‐acceptor assemblies with fullerenes,^[^ ^15^ ^]^ PbS QDs,^[^ ^16^ ^]^ ZnCdSeS QDs,^[^ ^17^ ^]^ and as hole‐transporting materials in perovskite solar cells.^[^ ^18^ ^]^ Pc aggregation is, however, the main loss channel for moderate power conversion efficiencies in dye‐sensitized solar cell schemes (DSSCs).^[^ ^19^ ^]^

Per se, either energy or charge transfer processes are expected in QD@phthalocyanine nanohybrids. But, attaching Pcs to the surface of QD goes often together with their aggregation. A non‐uniform surface coverage hampers analyses of energy or charge transfer processes and results in self‐quenching effects.^[^ ^17^ ^]^ Overcoming these issues requires minimizing aggregation without losing control over the formation of nanohybrids.

**Scheme 1:**
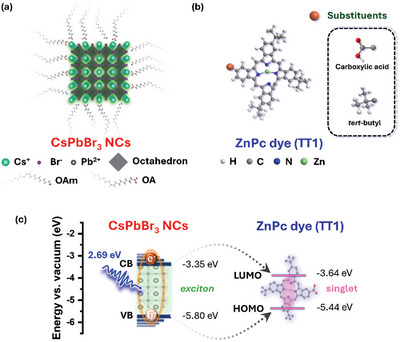
a) Structure of NCs that are passivated with organic ligands (OA: oleic acid and OAm: oleylamine) and b) molecular structures of unsymmetric ZnPc (carboxylic acid as anchoring group, TT1) and symmetric ZnPc_ref_ (tert‐butyl substituents). c) Energy level alignment between the NC and ZnPc against vacuum.

Herein, we illustrate selective energy transfer processes that evolve from CsPbBr_3_ perovskite NCs to surface‐bound ZnPcs (**Scheme** **1**), which facilitate disaggregation upon surface anchoring. Detailed time‐resolved analyses of the energy transfer processes in the NC@ZnPc nanohybrids were made possible using complementary spectroscopy techniques that range from femtoseconds to microseconds. First insights on emissive states and their lifetimes came from time‐resolved photoluminescence spectroscopy (TRPL). Then, femtosecond transient absorption spectroscopy (fs‐TAS) provided decisive information on the initial events, nanosecond transient absorption spectroscopy (ns‐TAS) enabled the detection of intermediate and non‐emissive states, and, finally, microsecond transient absorption spectroscopy (µs‐TAS) allowed to monitor long‐lived triplets.

It is only through the combination of these techniques that we reached a comprehensive understanding of the modus operandi when photoexciting NC@ZnPc and following its deactivation. We corroborated the first example of an unusual, concerted Dexter‐type singlet energy transfer using CsPbBr_3_ NCs as singlet photosensitizer of ZnPc dyes for singlet oxygen generation.

## 2 Results and Discussion

For the design of our electron donor‐acceptor nanohybrids, we considered two aspects. On the one hand, strong binding to immobilize the Pcs to the NC surface, and, on the other hand, an appropriate energy level alignment to facilitate energy or charge transfer processes. In this context, colloidal CsPbBr_3_ NCs (Scheme 1a) were prepared by the hot‐injection approach,^[^ ^20^ ^]^ and purified according to a modified strategy.^[^ ^7b^ ^]^ NCs with an average size of 6.5 ± 0.7 nm (Figure S1, Supporting Information; transmission electron microscopy, TEM analysis) and a surface‐to‐volume ratio that ensures sizeable interactions between NCs and ZnPcs were obtained.^[^ ^7^ ^]^

Unsymmetric Zn‐carboxyphthalocyanine (ZnPc or TT1, Scheme 1b) was synthesized according to Torres et al. by the direct transformation of hydroxy methyl phthalocyanine into its corresponding carboxyl derivative (TT1) catalyzed by ZnO in high yield. It was used as an energy acceptor.^[^ ^21^ ^]^ The presence of a carboxylic acid anchor guarantees its attachment to the NC surface as known for other NC@dye nanohybrids.^[^ ^4^, ^7^, ^21^, ^22^ ^]^ Scheme 1c summarizes the energy level alignment between the valence (VB, −5.80 eV) and conduction band (CB, −3.35 eV) of NCs with the highest‐occupied molecular (HOMO, −5.44 eV) and the lowest‐unoccupied molecular orbital level (LUMO, −3.64 eV) of ZnPc.^[^ ^7^, ^18^ ^]^ When considering such an energy band alignment, the Gibbs energy change for photoinduced charge transfer processes – either electrons and holes – renders thermodynamically feasible.

NC@ZnPc nanohybrids were prepared by titrating variable concentrations of ZnPc (2.5, 5.0, 7.5, and 10 µm) to an NC dispersion in toluene with a constant concentration (0.25 µm). This corresponds to NC:ZnPc ratios of 1:10; 1:20; 1:30 and 1:40, respectively. The optical properties of the as‐prepared nanohybrids were evaluated by spectroscopy techniques including steady‐state and time‐resolved absorption and photoluminescence spectroscopies.

### 2.1 Steady‐State Optical Properties and Evidence of Energy Transfer

**Figure 1:**
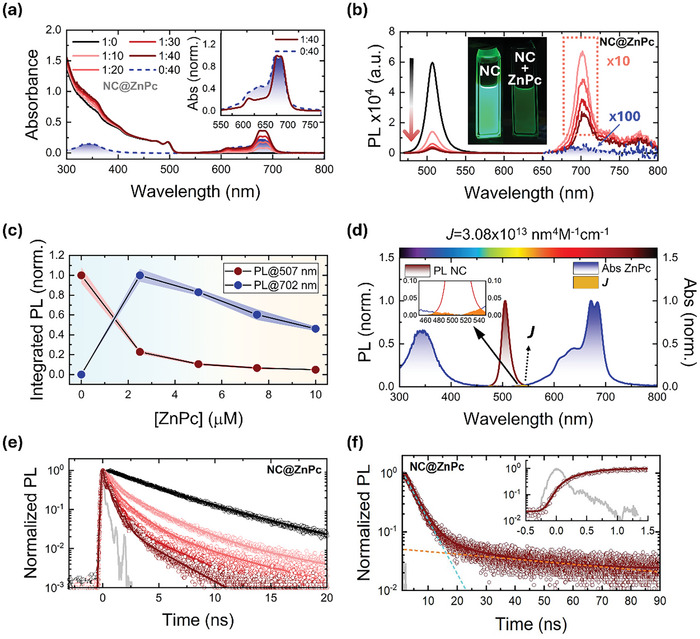
a) Steady‐state absorption spectra of NCs in the presence of variable concentrations of ZnPc [0–10 µm]. Inset: normalized absorption spectra of the Q‐band absorption of NC@ZnPc (1:40) and ZnPc. b) Steady‐state PL spectra of NCs in the presence of increasing concentrations of ZnPc [0–10 µm] under 460 nm photoexcitation. Photosensitized fluorescence of ZnPc in NC@ZnPc (red line) and intrinsic fluorescence of ZnPc (blue line) are multiplied by a factor of 10 and 100, respectively, due to their low emission in this spectral range. Inset: Digital photographs of NC and NC@ZnPc (1:40) under 365 nm photoexcitation. c) Evolution of the integrated PL as a function of ZnPc concentration at the NC and ZnPc maxima at 507 and 702 nm, respectively. d) Spectral overlap (*J*) between the normalized NC PL and ZnPc absorption. Inset: enlarged window. e) Time‐resolved PL of NCs in the presence of variable concentrations of ZnPc [0–10 µm] under 460 nm pulsed laser photoexcitation and 507 nm detection. f) Sensitized fluorescence of ZnPc in NC@ZnPc (1:40) at 702 nm under 460 nm pulsed laser photoexcitation. Inset: Early timescale of the sensitized ZnPc fluorescence in NC@ZnPc (1:40) illustrating the rise dynamics.

Standalone NCs featured an excitonic absorption at 496 nm, which is well in line with the NC size obtained by TEM analyses. As we increased the ZnPc concentrations, additional absorptions were discernable at 350 nm and in the 550–700 nm range, which corresponds to the Soret‐ and Q‐bands absorptions of ZnPc, respectively (**Figure** **1**a for the full spectra and Figure S2a, Supporting Information, range 550–800 nm).

NC@ZnPc absorbs a broader range of the solar spectrum than the individual components.^[^ ^23^ ^]^ Careful examination of the normalized absorptions of ZnPc and NC@ZnPc (Inset Figure 1a, NC:ZnPc ratio 1:40) reveals that, upon surface anchoring, the 637 nm shoulder decreases in intensity, and the highest‐energy Q‐band absorption shifts from 671 to 676 nm. Sizable electronic interactions in the ground state next to ZnPc disaggregation on the NC surface were derived by subtracting their absorptions (Figure S2, Supporting Information). Details regarding aggregation and disaggregation are given in Figures S3 and S4 (Supporting Information).

Emission spectra are displayed in Figure 1b following the selective NC photoexcitation at 460 nm. A drastic quenching of the NC‐centered emission was observed when increasing the ZnPc concentration. Concomitantly, the formation of a new 702 nm fluorescence band, assigned to the fluorescent singlet excited state (S_1_) of ZnPc (Figure 1c), was detected. It is noteworthy that the fluorescence of just ZnPc was subtle at this photoexcitation wavelength (Figure S5, Supporting Information). This points to an NC‐to‐ZnPc energy transfer photosensitization. Already at low ZnPc concentrations of <2 µm (NC:ZnPc of 1:50; Figure S6a,b, Supporting Information) the NC emission is efficiently quenched. An association constant (*K*_app_) of 1.4 × 10^6^ m ^−1^ was calculated from the NC‐centered emission quenching at different concentrations of ZnPc (Figure S6c, Supporting Information), which confirmed the strong binding of ZnPc to the NC surface.

We performed additional ATR‐FTIR measurements to confirm the attachment of ZnPc to the NC surface. With the help of Figure S7 (Supporting Information), we have documented the disappearance of the O─H stretching of the ─COOH anchor at 3384 cm^−1^, and the shift of the C═O stretching of the carboxylic acid from 1697 to 1536 cm^−1^.^[^ ^23^ ^]^ These findings confirm that all ZnPc are anchored to the NC surface. Moreover, the C═O stretching at 1641 cm^−1^ stems from oleate bound to the NC surface.^[^ ^7a^ ^]^

Dynamic light scattering and Zeta potential measurements were performed for the NC and the NC@ZnPc nanohybrid (Figure S8, Supporting Information). An increase of 2.2 nm has been noticed in the hydrodynamic radius of NC (6.1 nm) compared to NC@ZnPc (8.3 nm). This agrees quite well with the ZnPc incorporation into the organic shell. FTIR spectroscopy has demonstrated this independently (Figure S7, Supporting Information). Moreover, a slight change in the surface charge suggests the replacement of oleic acid ligands by ZnPc roughly maintains the electroneutrality of the NC surface in NC@ZnPc. It is noteworthy that previously reported binding constants of organics to perovskite NCs are lower, for instance, 5.2 × 10^4^ m ^−1^ for Rhodamine,^[^ ^5a^ ^]^ and 2 × 10^5^ m ^−1^ for cyanine.^[^ ^6^, ^24^ ^]^

Excitation spectra were recorded for different NC:ZnPc ratios (1:10–1:40) at the 702 nm emission maximum and compared to that of ZnPc (Figure S10a,b, Supporting Information). We take the presence of the perovskite features in the excitation spectra of NC@ZnPc as confirmation for the formation of (S_1_) of ZnPc by means of photosensitization by NCs. The energy transfer efficiency from the energy‐donating NCs to the energy‐accepting ZnPc was estimated by comparison of the normalized excitation and absorption spectra to the absorption maximum of ZnPc (Figure S10c–f, Supporting Information) reaching values of ≈45%. Figure S11 (Supporting Information) illustrates the energy transfer efficiency across the concentration range of ZnPc all the way up to 10 µm.

Reaching a plateau at 5 µm with 45% suggests that non‐emissive processes compete with energy transfer. Control experiments were performed with a ZnPc reference (ZnPc_ref_) that lacks any anchoring groups (Scheme 1b). ZnPc_ref_ revealed similar absorption features as ZnPc, with a Q‐band absorption between 550–750 nm and a Soret‐band absorption at 350 nm (Figure S12a, Supporting Information). This finding confirms that the electronic structure of ZnPc is unaffected by the substitution with *tert*‐butyl groups. The emission spectra of NCs were not affected by the presence of any ZnPc_ref_ up to an NC:ZnPc_ref_ ratio of 1:50 (Figure S12b, Supporting Information). More importantly, no sensitized ZnPc_ref_ fluorescence was observed (Figure S12c, Supporting Information). Excitation spectra, which were taken at 702 nm, correspond exclusively to the excitation spectra of ZnPc_ref_ without any features from NCs (Figure S12d, Supporting Information).

This observation confirms the fundamental role of the anchoring group. It enables the ZnPc attachment onto NC surfaces, which provides a through‐bond Dexter‐type pathway for an NC‐to‐ZnPc energy transfer. By virtue of a favorable band alignment (Scheme 1c) and a high association constant (Figure S6c, Supporting Information), several mechanisms might be operative in the emission quenching in NC@ZnPc. On one hand, it might be a Dexter‐ or Förster‐type singlet energy transfer from NC to ZnPc or a sequential electron and hole transfer from NC to ZnPc. A negligible spectral overlap integral (*J*) between NC emission and ZnPc absorption of 3.1 × 10^13^ nm^4^ m ^−1^ cm^−1^ (Figure 1d) prompts that FRET is unfavored. To gather a comprehensive understanding of the energy transfer mechanism, TRPL and TAS were employed across various time scales that range from femtoseconds to microseconds.

### 2.2 Time‐Resolved Photoluminescence Spectroscopy

Recombination from band‐edge states of NCs were analyzed by means of time‐correlated single photon counting (TCSPC) experiments with NCs and NC@ZnPc nanohybrids (NC:ZnPc ratios increasing from 1:10 to 1:40) using excitation and emission wavelengths of 460 and 507 nm, respectively (Figure 1e).^[^ ^25^ ^]^ NC emission is frequently based on a single excitonic state together with a set of closely spaced fine‐structure states, which are populated in accordance with a thermal distribution function.^[^ ^26^ ^]^ The NC emission decay was well‐fitted to a triexponential decay function. The corresponding lifetimes along with their relative contributions are 2.1 ns (16%), 5.0 ns (76%), and 17.7 ns (8%) as shown in Table S1 (Supporting Information). The short‐ and long‐lived components are associated with shallow (STX) and deep (DTX) trap‐mediated exciton recombinations, respectively, while the intermediate‐lived component arises from direct electron‐hole exciton recombinations (X).^[^ ^27^ ^]^ Implicit are surface defects and multiple trapping and detrapping of charge carriers that come along with electron‐hole recombinations.^[^ ^28^ ^]^ Consistent with this interpretation is the fact that the time‐resolved emission spectrum (TRES) afforded the same 507 nm maximum excitonic emission (Figure S13, Supporting Information).

Notably, all three‐time components gradually shortened in the presence of increasing concentrations of ZnPc (Table S1, Supporting Information). For NC@ZnPc with an NC:ZnPc ratio of 1:40, the TCSPC lifetimes are 0.3 ns / 65% (STX), 1.9 ns / 30% (X), and 7.9 ns / 5% (DTX). As such, all of them are involved in the interfacial energy transfer with energy transfer rate constants (*k*_ET_) of 2.6 × 10^9^, 0.3 × 10^9^, and 0.1 × 10^9^ s^−1^ for (STX), (X), and (DTX), respectively (see Supporting Information for rate constants and quantum yields calculation). Furthermore, taking the TCSPC data we calculated the corresponding quantum yields (*Φ*_ET_) as 84% (STX), 61% (X), and 55% (DTX). The highest *Φ*_ET_ obtained for STX underlines the crucial role of surface states in mediating interfacial processes, which could be applicable in photon upconversion, photocatalysis, and optoelectronics.^[^ ^29^ ^]^ On this basis, NC‐to‐ZnPc energy transfer takes place on three distinct timescales: below 1 ns, between 1–5 ns, and beyond 10 ns (see Supporting Information for calculation on time constants).

To monitor the ZnPc (S_1_) fluorescence, excitation, and emission detectors were set to 460 and 702 nm, respectively (Figure 1f). For ZnPc, just a monoexponential decay evolved with a 2.8 ns lifetime. Selective NC excitation in NC@ZnPc at 460 nm afforded three contributions for the ZnPc (S_1_) fluorescence: 0.6, 3.7 (79%), and 22.3 ns (21%). What is of great value is that the 0.6 ns component is seen as a rise in the fluorescence intensity. Its decay is intrinsic with a lifetime of ≈2.8 ns. This corroborates the NC‐to‐ZnPc energy transfer and the rate constant of 2.6 × 10^9^ s^−1^ (See Supporting Information for further details). Energy transfers from (X) with 3.7 ns and (DTX) with 22.3 ns are slower than the intrinsic (S_1_) decay of 2.8 ns. Consequently, they are only observed as delayed fluorescence decays similar to that reported for an activated reverse intersystem crossing.^[^ ^30^ ^]^ A Förster‐type energy transfer, with an estimated rate constant for the STX component of 0.17 × 10^9^ s^−1^, that is, ≈15 times slower than the experimentally determined 2.6 × 10^9^ s^−1^, is, however, highly unlikely (see Supporting Information for calculation on time constants).

### 2.3 Femtosecond Transient Absorption Spectroscopy

fs‐TAS was used to probe the ultrafast excited‐state dynamics. For NCs, a 460 nm photoexcitation (2.69 eV) and a pump energy of 1 µJ was utilized. Considering an NC band gap energy of 2.49 eV (Figure S14, Supporting Information), an energy excess of 0.2 eV translates into a higher electronic excited state formation. In Figure S16a (Supporting Information), which shows differential absorption spectra at given time delays, several transient features are detectable. Early on, namely, 1 ps, exciton bleaching (XB) is observed at 477 and 498 nm next to excited‐state absorptions (ESA) below 450 and at 515 nm. This is due to state‐filling effects at the edges of valence as well as conduction bands due to forming high‐energy states and Coulomb interactions between electron‐hole pairs created by the pump and probe pulse, respectively. In the next 10 ps, suppression of the 477 nm XB and 515 nm ESA next to a red shift of the ESA in the 430–460 nm range is noted. This is followed within the next 25 ps by a narrowing of the XB along with an attenuation of the 498 nm XB and 450 nm ESA. At delay times longer than 100 ps, the 498 nm XB feature progressively decays. The evolution at the early time window (1–10 ps) is assigned to the cooling of exciton/charge carriers. Here, sub‐bandgap features (515 nm) are replaced by the 498 nm XB. This happens throughout the cooling of high‐energy charge carriers down to band‐edge states.^[^ ^31^ ^]^ At long delay times (>100 ps), the XB decay corresponds to radiative exciton recombination as observed in TCSPC. Detailed discussions of the decay are gathered in the Supporting Information. In Figure S17a (Supporting Information), we gathered fs‐TAS experiments for NC following 460 nm photoexcitation with a pump fluence of 1 µJ. 498 and 688 nm probe wavelengths were used for probing XB of NC and, when present, ground‐state bleaching (GSB) of ZnPc, respectively (Figure S17b, Supporting Information).

A deeper understanding of the underlying processes came from global analyses of the data using a target kinetic model.^[^ ^32^ ^]^ Global analysis of NC required the use of five species (Figure S17c and Table S2, Supporting Information). Time constants of 5.8, 11, and 34 ps are ascribed to hot excitons (hot‐X), multiexcitons (MX), and biexcitons (XX), respectively. Additional time constants of 1.0 and 4.4 ns correspond to (STX) and (X), respectively. All of this is accompanied by an infinite offset accounting for the long‐lived exciton recombination. Under our experimental conditions involving high‐pump energies, intra‐band cooling occurs on the picosecond timescale, as previously observed.^[^ ^33^ ^]^ Many‐body interactions such as (XX) and (MX) also participate in the decay cascades.^[^ ^34^ ^]^ When turning to (hot‐X), its lifetime of 5.8 ps is rather long. We rationalize this by the high pump energies of 1 µJ. At lower pump energies of 0.15 µJ, it gets as short as 0.3 ps (Figure S16d, Supporting Information) and it is similar to that found in the literature.^[^ ^35^ ^]^ The differential absorption changes for (MX) were recorded at low (0.15 µJ) and high (1 µJ) pump energies (Figure S16e, Supporting Information). Overall, the lifetime was shortened from 11 ps at 1 µJ to 3.2 ps at 0.15 µJ and the intensity of the 525 nm ESA was lower at 0.15 µJ than at 1 µJ. This agrees with the emergence of (MX) at high pump fluences as previously reported.^[^ ^36^ ^]^ Furthermore, the differential absorption spectrum of (XX) resembles those reported for similar NC sizes.^[^ ^26^, ^37^ ^]^

fs‐TAS of ZnPc under 670 nm photoexcitation (1 µJ) showed GSB and stimulated emission (SE) superimposed with a broad ESA across the visible range (Figure S18, Supporting Information). Dynamics evolved along two exponentials of 16 ps and 1.9 ns. These correspond to the vibrationally‐hot S_1_ state (hot‐S_1_) and vibrationally‐relaxed state (S_1_), respectively, and are accompanied by an infinite offset ascribed to the triplet excited state (T_1_) population (Table S2, Supporting Information). The shape of the vibrationally hot (S_1_) is quite like that of the vibrationally relaxed (S_1_), suggesting the underlying relaxation involves the same electronically excited state. Similar vibrational cooling times have been reported for related phthalocyanines.^[^ ^38^ ^]^

**Figure 2:**
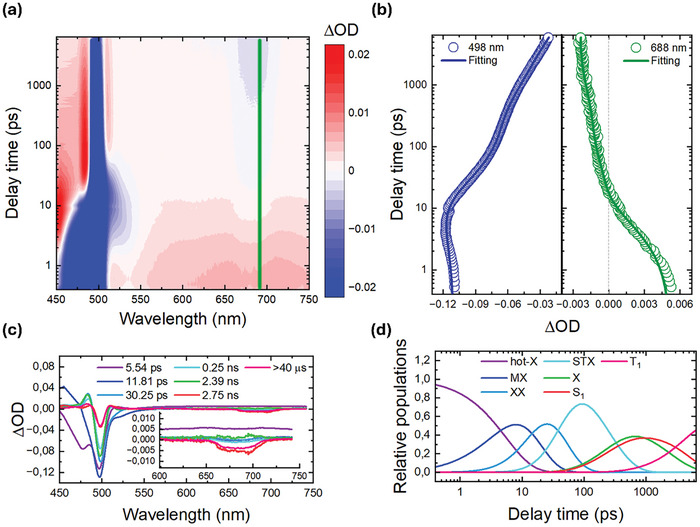
fs‐TAS of NC@ZnPc (1:40) nanohybrid under 1 µJ pump excitation at 460 nm. a) 2D pseudo‐color plot of raw data for 0.5 ps–7.5 ns timescale. b) Representative decay traces and multiexponential fittings at 498 and 688 nm for XB of NC and GSB of ZnPc, respectively. c) Species‐associated differential spectra (SADS) obtained by target analysis for hot‐X (5.54 ps), MX (11.81 ps), XX (30.25 ps), STX (0.25 ns), X (2.39 ns), S_1_ (2.75 ns) and T_1_ (>40 µs). d) Corresponding relative populations for the species indicated in (c).

The situation is quite different when NC@ZnPc were photoexcited at 460 nm. A decay of all NC features was observed concomitantly with the delayed growth of the 688 nm GSB of ZnPc (**Figure** **2**a). A kinetic inspection revealed that the transformation took <1 ns (Figure 2b). The global analysis resulted in six species and an infinite offset (Figure 2c,d and Table S2, Supporting Information). The three shortest‐lived species with lifetimes of 5.5, 12, and 30 ps, were ascribed to (hot‐X), (MX), and (XX), respectively. None of them showed sizeable changes when compared to NCs and, in turn, none of them contributed to any energy transfer. Turning to the three longest‐lived species, their lifetimes of 0.25, 2.4, and 2.8 ns were ascribed to (STX), (X), and (S_1_), respectively. However, only the earlier two were found to be shortened and, in turn, confirmed their involvement in an energy transfer. Critical is the fact their lifetimes match those determined in TCPSC (Figure 1e and Table S1, Supporting Information).^[^ ^39^ ^]^ The lack of any of the fingerprints known for either the one‐electron oxidized or the one‐electron reduced forms of ZnPc in fs‐TAS demonstrates the absence of sequential electron/hole transfer pathways and indicates an exclusive concerted energy transfer pathway.

### 2.4 Nanosecond Transient Absorption Spectroscopy

ns‐TAS provided additional details about the energy transfer but on longer time scales (>10 ns). For NCs, using a four‐species kinetic model is deemed necessary. Global analysis of the data afforded time constants of 2.1, 4.9, and 17.7 ns for (STX), (X), and (DTX), respectively. We note a reasonable agreement with those values observed in TCSPC (Figure S19, Supporting Information). A fourth species of ≈100 ns lifetime was assigned as a dark trap state (DT). (DT) is, however, non‐emissive and responsible for the moderate quantum yield (*Φ*_PL_) of 45%. For ZnPc upon 670 nm photoexcitation, two exponential decays were observed. 2.6 and 690 ns relate to (S_1_) and (T_1_), respectively (Figure S20, Supporting Information).

**Figure 3:**
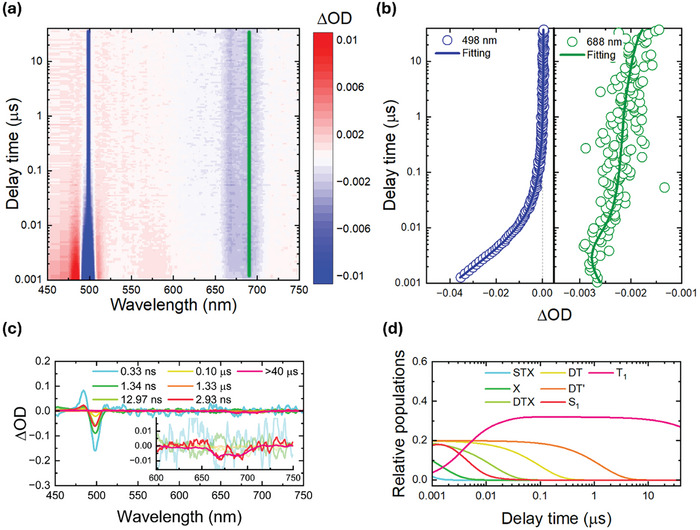
ns‐TAS of NC@ZnPc (1:40) nanohybrid under 1 µJ pump excitation at 460 nm. a) 2D pseudo‐color plot of raw data for 1 ns–40 µs timescale. b) Representative decay traces and multiexponential fittings at 498 and 688 nm for XB of NC and GSB of ZnPc, respectively. c) SADS obtained by target analysis for STX (0.33 ns), X (1.34 ns), DTX (12.97 ns), DT (0.1 µs), DT’ (1.33 µs), S_1_ (2.93 ns) and T_1_ (>40 µs). d) Corresponding relative populations for the species indicated in (c).

When analyzing NC@ZnPc, a complex evolution was observed upon photoexcitation (**Figure** **3**a,b). Altogether, seven species were required to fit the experimental data (Figure 3c,d).

**Figure 4:**
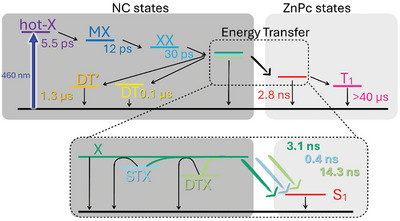
A full picture of all involved states after selective NC excitation and corresponding singlet sensitization mechanism from three excitonic components (X: direct electron‐hole exciton recombination, STX: shallow trap mediated exciton recombination, and DTX: deep trap mediated exciton recombination) of NCs to the ZnPc singlet excited state (S_1_), followed by the triplet excited state formation (T_1_).

The branching model in **Figure** **4**, includes, on one hand, three excitonic species, that is, (STX), (X), and (DTX), plus the dark (DT) of NCs and, on the other hand, (S_1_) and (T_1_) of ZnPc. An additional dark trap (DT’) state with a 1.3 µs lifetime was registered (see Figure S21, Supporting Information for the species‐associated differential spectra), which stems from surface defects that are associated with the loss of surface ligands during exchange with ZnPc.^[^ ^40^ ^]^ Notable is also the fact that the (*T*_1_) lifetime of ZnPc increases from 690 ns to >40 µs due to ZnPc disaggregation on the NC surface. A similar observation has previously been forwarded for zinc 2,11,20,29‐tetra‐*tert*‐butyl‐2,3‐naphthalocyanine solids.^[^ ^38^ ^]^

### 2.5 Microsecond Transient Absorption Spectroscopy

Microsecond transient absorption spectroscopy (µs‐TAS) was used to evaluate the performance of the triplet excited state of ZnPc in the NC@ZnPc. Upon 460 nm photoexcitation of NC, where ZnPc negligibly absorbs, we identified (T_1_) with its 485 nm ESA, which was absent in the reference experiments (Figure S22, Supporting Information). It is noteworthy that the ZnPc (T_1_) lifetime in NC@ZnPc (Figure S22c, Supporting Information) was longer (8.0 µs) than that of (T_1_) in ZnPc reference (Figure S22d, Supporting Information) when photoexcited at 620 nm (4.5 µs). This agrees well with the trend observed in the ns‐TAS measurements. Selective ZnPc photoexcitation at 620 nm enhanced the (T_1_) yield in NC@ZnPc due to ZnPc disaggregation (Figure S22e, Supporting Information). For example, at a 1:40 ratio in NC@ZnPc, the (T_1_) yields were amplified by a factor of 1.3 using the same optical density at the excitation wavelength.

When molecular oxygen was added (Figures S22c and S23c, Supporting Information), (T_1_) of ZnPc in NC@ZnPc was quenched in an intermolecular fashion. To further shed light on that, we recorded singlet oxygen (^1^O_2_) in phosphorescence measurements at 1277 nm (Figures S22f and S23f, Supporting Information). In the case of NC@ZnPc, sizeable (^1^O_2_) phosphorescence under 460 nm photoexcitation is in stark contrast to the lack of it for the ZnPc reference. Under 620 nm photoexcitation, the difference is as large as 1.7 for NC@ZnPc and ZnPc. These findings underline the potential of NC@ZnPc to sensitize the formation of (^1^O_2_). Clearly beneficial are lifetimes from a longer (T_1_) lifetime.

The photostability of the NC@ZnPc nanohybrid was evaluated under continuous light excitation from 445 to 465 nm and 630 to 650 nm and the absorption spectra before and after the irradiation were recorded (Figure S24, Supporting Information). Although the NC in the nanohybrid was photostable under both excitations, the ZnPc was photo‐unstable when light from 445 to 465 nm was employed. To address any instabilities, introducing substituents on the phthalocyanine core or preparing Ni‐based phthalocyanines (NiPc) seems like reasonable alternatives.^[^ ^41^ ^]^

### 2.6 Mechanistic Aspects

With the aforementioned information at hand, we were able to disclose the full picture of the excited‐state decay cascade in NC@ZnPc based on a singlet energy transfer via a Dexter mechanism (Figure 4). After NC photoexcitation, (X) is generated in a sequential fashion. From this point on, the exciton population branches, feeding dark states, on the one hand, and undergoing energy transfer, on the other hand. All excitonic and (S_1_) species in TAS and TCSPC measurements were consistent with each other. Importantly, energy transfer from NC to ZnPc in NC@ZnPc (2.6 × 10^9^ s^−1^) is faster than that reported for ZnCdSeS QDs@ZnPc (5.8 × 10^8^ s^−1^). Overall, the measured rate constant was one order of magnitude larger than that calculated based on the classical Förster‐type model (see Supporting Information for further details).^[^ ^17^, ^42^ ^]^

Perovskite NCs have been used as efficient (S_1_) and (T_1_) photosensitizers for energy‐accepting dyes that are anchored to their surface.^[^ ^4^ ^]^ For all known examples of singlet sensitization, either a Förster‐type energy transfer or sequential Dexter‐like electron–hole transfers is operative. For example, (S_1_) transfer from CsPbBr_3_ NC to pentacene was mediated by an ultrafast hole transfer (5 ps) followed by a slower electron transfer (400–600 ps) before singlet fission took place.^[^ ^11^ ^]^ However, when Rhodamine was used as an energy acceptor, the energy transfer between CsPbBr_3_ and Rhodamine‐B dye was mediated by a Förster‐type mechanism with a rate constant of 5.9 × 10^10^ s^−1^. The introduction of chloride in the NC led to an additional energy‐transfer pathway via the Dexter‐type mechanism.^[^ ^5a^ ^]^ In the case of 4‐carboxyphenyl‐BODIPY as energy acceptor, Förster‐type energy transfer was observed with 3.8 × 10^10^ s^−1^, while an ultrafast hot hole transfer (1 × 10^12^ s^−1^) dominated when iodine‐BODIPY was used.^[^ ^7b^ ^]^

The effect of the temperature on the energy transfer dynamic was evaluated. The rise in the sensitized ZnPc (S_1_) was slowed down from 0.38 to 0.64 ns upon increasing the temperature from 10 to 30 °C. At high temperatures, additional processes start to compete with energy transfer, such as enhanced nonradiative pathways, ZnPc detaching from the NC surface, and domination by alternative kinetic and entropy factors. The complex interplay between all these competitive processes seems to contribute to the negative activation energy (*E*_a_) of −0.41 eV, calculated from the Arrhenius plot slope (Figure S25, Supporting Information).

It is noteworthy that anchoring of ZnPc to the NC surface results in the first example of concerted (S_1_) transfer via Dexter using CsPbBr_3_ NCs as visible light‐harvesting antenna, with the generation of singlet oxygen as proof‐of‐concept application, using a comprehensive analysis across relevant timescales, as can be summarized in Figure S26, Supporting Information. The following findings support the Dexter singlet energy transfer as the unique operative mechanism: i) negligible spectral overlap integral (*J*) of 3.1 × 10^13^ nm^4^ m ^−1^ cm^−1^ between CsPbBr_3_ and ZnPc; ii) lower estimated Förster‐type singlet energy transfer (0.17 × 10^9^ s^−1^) than the experimentally determined rate constant of 2.6 × 10^9^ s^−1^ obtained from TCSPC and TAS experiments; iii) adequate energy levels to drive a concerted charge transfer event; iv) absence of evidence of the formation of anion/cation radicals in transient spectroscopy studies; v) strong electronic coupling as a consequence of molecular planarity and hydrophobicity; and vi) energy transfer was only observed when a carboxylic acid is tethered to the phthalocyanine, which ensure the close contact between energy donors and acceptors to establish orbital overlap.

## 3 Conclusion 

In summary, a comprehensive analysis of the excited state dynamics of NC and ZnPc in NC@ZnPc nanohybrids was conducted with the help of an arsenal of experimental techniques. Hereby, steady‐state measurements were complemented by time‐resolved absorption and emission spectroscopies. The main findings are summarized as follows. First, CsPbBr_3_ NCs act as an efficient singlet photosensitizer of surface‐anchored ZnPcs. Second, three NC excitonic states are involved in the energy transfer process. Third, an unusual, concerted Dexter‐type singlet energy transfer is the unique mechanism with an upper‐limit efficiency of 45%. Fourth, the surface binding of ZnPc provokes its disaggregation and creates dark NC trap states that open a nonradiative pathway and decrease the energy transfer efficiency.

Our results pave the way for innovations in energy harvesting and conversion technologies. The ability to control and prolong triplet‐excited state lifetimes of photoactive dyes and their use to photosensitize the formation of singlet oxygen opens new avenues for the development of new advanced materials with improved performance of light‐energy conversion in triplet energy transfer photocatalysis and photovoltaic devices that require efficient charge separation.

## Conflict of Interest

The authors declare no conflict of interest.

## Supporting Information

Supporting Information is available from the Wiley Online Library or from the author. Corresponding materials, methods, instrumentation, additional figures, and tables. The authors have cited additional references within the Supporting Information.^[^ ^7^, ^15^, ^19^, ^20^, ^23^, ^26^, ^31^, ^32^, ^36^, ^37^, ^43^ ^]^

## Supporting information



Supporting Information

## Data Availability

The data that support the findings of this study are available from the corresponding author upon reasonable request.
