# Supplementary material for: Deciphering the Energy Transfer Mechanism Across Metal Halide Perovskite‐Phthalocyanine Interfaces
Source: Adv Sci (Weinh). 2025 Jan 10;12(9):2414831. doi: 10.1002/advs.202414831 (PMC11884585; doi:10.1002/advs.202414831)
Supplement: Supplementary file 1 — Supporting Information [file ADVS-12-2414831-s001.docx]

Supporting Information

Deciphering the Energy Transfer Mechanism Across Metal Halide Perovskite-Phthalocyanine Interfaces

Alejandro Cortés-Villena, Alejandro Cadranel, Kobra Azizi, Tomás Torres,* Dirk M. Guldi,* Julia Pérez-Prieto,* and Raquel E. Galian*

|  |  |  |
| --- | --- | --- |
| **Table of Contents** | |  |
| **Materials** | |  |
| Perovskite nanocrystals | |  |
| **Methods** | |  |
| Synthesis of CsPbBr_3_ perovskite nanocrystals | |  |
| **Instrumentation** | |  |
| **Calculation of energy transfer rate constants (*k*_ET_)** | |  |
| **Calculation of energy transfer time constants (τ_ET_)** | |  |
| **Calculation of energy transfer quantum yields (*Φ*_ET_)** | |  |
| **Estimation of theoretical energy transfer rate constants (*k*_ET_)_t_** | |  |
| **Figure S1.** TEM image of NCs and NC@ZnPc. | |  |
| **Figure S2.** Zoom in of absorption spectra of NC at increasing ZnPc concentration [0-10 μM]. Difference spectra and resultant electronic interaction component from subtraction. | |  |
| **Figure S3.** Absorption spectra of ZnPc and NC@ZnPc and Lambert-Beer relationship at increasing ZnPc concentrations [0-10 μM]. | |  |
| **Figure S4.** Absorption spectra of ZnPc at increasing NC concentration [0-30.3 nM]. | |  |
| **Figure S5.** PL spectra of ZnPc and normalized PL of NC@ZnPc under 460 nm. | |  |
| **Figure S6.** PL spectra of NCs and NC@ZnPc at 10 μM ZnPc under 460 nm. PL evolution at increasing ZnPc concentration [0-2 μM]. Double-reciprocal analysis of the PL quenching. | |  |
| **Figure S7.** ATR-FTIR spectra of the ZnPc and NC@ZnPc (1:40) nanohybrid. | |  |
| **Figure S8.** Size distribution and Zeta potential for NC and NC@ZnPc (1:40) nanohybrid. | |  |
| **Figure S9.** PL spectra of ZnPc and NC@ZnPc under 670 nm at increasing ZnPc concentration [0-2 μM]. | |  |
| **Figure S10.** PLE spectra of NC at increasing ZnPc concentration [0-10 μM]. | |  |
| **Figure S11.** Energy transfer efficiency of NC@ZnPc nanohybrids. | |  |
| **Figure S12.** Control experiments with the ZnPc_ref_ dye. Absorption, PL and PLE spectra of ZnPc_ref_ and NC+ZnPc_ref_. | |  |
| **Figure S13.** TRES map of NCs under 460 nm recorded between 480 and 550 nm. | |  |
| **Figure S14.** Second derivative of the NC absorption spectrum.  **Figure S15:** Fluence-dependence fs-transient absorption probed at XB. | |  |
| **Figure S16.** Power-dependent TAS studies. | |  |
| **Figure S17.** fs-TAS experiments on NCs under 460 nm and target analysis. | |  |
| **Figure S18.** fs-TAS experiments on ZnPc under 670 nm and target analysis. | |  |
| **Figure S19.** ns-TAS experiments on NCs under 460 nm and target analysis. | |  |
| **Figure S20.** ns-TAS experiments on ZnPc under 670 nm and target analysis. | |  |
| **Figure S21.** SADS extracted from target analysis. | |  |
| **Figure S22.** μs-TAS and singlet oxygen experiments under 460 nm. | |  |
| **Figure S23.** μs-TAS and singlet oxygen experiments under 620 nm.  **Figure S24.** Absorption spectra before and after irradiation at different wavelengths.  **Figure S25.** Arrhenius plot for the ZnPc (S1) formation rate constant at variable temperatures.  **Figure S26:** Combination of all photophysical processes involved in the system represented for the NC@ZnPc nanohybrid. | |  |
| **Table S1.** Time constants from TCSPC. | |  |
| **Table S2.** Rate and time constants from fs-TAS obtained by target analysis. | |  |
| **Table S3.** Rate and time constants from ns-TAS obtained by target analysis. | |  |

**Materials**

**Perovskite nanocrystals**

Cesium carbonate (Cs_2_CO_3_, 99 %, Sigma-Aldrich), oleic acid (OA, 90 %, Alfa Aesar), 1-octadecene (1-ODE, 90 %, Alfa Aesar), lead bromide (PbBr_2_, 99.999 %, Sigma-Aldrich), oleylamine (OAm, 70 %, Sigma-Aldrich), toluene (C_7_H_8_, 99.5 %, Scharlau) and methyl acetate (MeOAc, 99.5 %, Sigma-Aldrich).

**Methods**

**Synthesis of CsPbBr_3_ perovskite nanocrystals**

The CsPbBr_3_ perovskite nanocrystals (NCs) were synthesized accordingly by a previously reported *hot-injection* method with slight modifications.^[7b, 20]^ All experiments were carried out using a Schlenk line. First of all, a solution of cesium oleate (0.125 M in 1-ODE) was prepared by mixing cesium carbonate (407 mg, 1.24 mmol) and oleic acid (1.25 mL, 3.53 mmol) in 1-octadecene (20 mL) in a 50 mL three-neck round-bottom flask and the mixture was degassed under vacuum (0.49 mbar) at 120 ˚C for 1 h. Then, the mixture was heated up to 150 ˚C under N_2_ atmosphere until Cs_2_CO_3_ was completely dissolved (ca. 30 min) obtaining a clear solution. Afterwards, the solution was slowly cooled down to room temperature and stored under N_2_ atmosphere for being used. On the other hand, for the synthesis of NCs, lead bromide (72 mg, 0.196 mmol), oleylamine (0.71 mL, 1.5 mmol) and oleic acid (0.53 mL, 1.5 mmol) were loaded into a 50 mL three-neck round-bottom flask with 1-octadecene (4.8 mL) and degassed under vacuum (1 mbar) at 100 ˚C for 30 min. Then, the valve of the Schlenk line was changed to N_2_ flow and after 15 min, it yielded a colorless solution. At the same time, the cesium oleate solution was heated up to 120 ˚C under N_2_ atmosphere. After complete solubilization of the lead bromide salt, the reaction temperature was raised to 190 ˚C and cesium oleate solution (0.6 mL, 0.075 mmol) was swiftly injected through a purged syringe and, 5 s later, the reaction was quenched through an ice/water bath (bath temperature ca. -2 ˚C) and the NCs were finally subjected to purification. The crude dispersion was first diluted with 5 mL of toluene and transferred to a 50 mL centrifuge tube to initiate the isolation process. The crude was first centrifuged at 2500 rpm for 10 min (15 ˚C). The supernatant was carefully pipetted out and transferred to another 50 mL centrifuge tube. Methyl acetate anhydrous (8 mL) was added to the supernatant to help the precipitation of smaller NCs and was secondly centrifuged at 5000 rpm for 10 min (15 ˚C). This second precipitate was finally redispersed in 2 mL of toluene. This dispersion was finally filtrated out through a 0.2 μm membrane filter to yield highly colloidal, homogeneous CsPbBr_3_ NCs dispersion.

**Instrumentation**

**Steady-state UV-vis-NIR absorption spectroscopy.** Steady-state UV-vis-NIR absorption spectra were recorded on a UV/Vis/NIR Perkin Elmer Lambda 1050 spectrophotometer equipped with deuterium and tungsten halogen light sources and a photomultiplier tube (PMT) detector covering from 300 to 800 nm.

**Steady-state photoluminescence spectroscopy.** Stationary photoluminescence (PL) spectra (emission and excitation) were recorded on a FLS1000 photoluminescence spectrometer (Edinburgh Instruments) equipped with a 450 W ozone free continuous xenon arc lamp and a photomultiplier (PMT-980) detector in cooled housing with extended spectral range from 250 to 800 nm. A 460 and 670nm excitation wavelength was used for selectively excite the NCs and ZnPc dye.

**Photoluminescence quantum yield.** Absolute photoluminescence quantum yields (*Φ*_PL_) were recorded on a FLS1000 photoluminescence spectrometer equipped with an integrating sphere system with a reflectance higher than 99% in the range 400-800 nm. Sample solutions with 0.1 OD at 460nm excitation wavelength was used to minimize re-absorption effects. For reference, the neat solvent with exactly the same volume was used.

**Time-resolved photoluminescence spectroscopy.** Time-resolved photoluminescence (TRPL) measurements were recorded on a FLS1000 photoluminescence spectrometer through the time-correlated single photon counting (TCSPC) technique coupled with a 460 nm picosecond pulsed supercontinuum white light laser (SuperK, repetition rate: 9.8MHz, NKT Photonics) and a microchannel plate (MCP-900) detector in cooled housing. A Ludox solution (0.1 OD at excitation wavelength) was used as instrument response function (IRF). The IRF is about 200 ps in our setup.

**Femto- and nanosecond transient absorption spectroscopy.** Ultrafast transient absorption experiments were conducted using an Astrella-F-1K amplified Ti:sapphire femtosecond laser system from Coherent, operating at a repetition rate 1kHz, 5.5 W power (5 mJ pulse energy), pulse duration of 80 fs, with TA pump / probe Helios and EOS detection systems from Ultrafast Systems. White light was generated focusing a fraction of the fundamental 800 nm output onto a 2 mm sapphire crystal (Helios). A 1.2 mJ fraction of the fundamental is used for pump beam generation by a TOPAS Prime from Light Conversion with standard NirUVis extension. The pump energy was varied between 150 and 1000 nJ. The laser spot diameter was estimated to be 0.5 cm. Therefore, assuming a spherical shape, the area was ca. 0.196 cm^2^. The calculated energy fluence was 5.1 μJ/cm^2^ for 1 μJ. A depolarizer was placed in the pump beam to avoid rotational dynamics. Bandpass filters with ± 5 or ± 10 nm were used to ensure low spectral width and to exclude 800 nm photons. All measurements were conducted in a 2 mm quartz cuvette under argon atmosphere, using solutions with absorbances of 0.5-0.7 under continuous stirring. To analyze transient absorption data, we used a suggested procedure.^[32a]^ We start with SVD and global analysis, using an all-sequential decay model that provides evolution associated spectra of potentially intervening species, to determine the number of decaying species that participate in the decay cascade. However, this does not necessarily yield differential spectra with genuine physicochemical meaning. Afterwards, a target analysis is applied, using specific target models that result in species associated difference spectra (SADS) with true physicochemical meaning. Obtained data were treated by SVD, global and target analyses using the R- package TIMP and GloTarAn.^[32, 43a]^

**Microsecond transient absorption spectroscopy.** Microsecond transient absorption spectroscopy (μs-TAS) employing spectral and time-resolved transient species absorption measurements were performed on a laser flash photolysis spectrometer (LP980-KS, Edinburgh Instruments) equipped with a Quanta-Ray INDI Nd:YAG laser (Spectra Physics), with a parametric optical oscillator (primoScan BB, Spectra Physics). The laser output energy was 5 and 1 mJ for the pumping 460 and 620 nm wavelengths, and the instrumental response function was 10 ns. Spectral measurements were made using an ICCD camera (Andor DH320T) that integrates for 500 ns (gate width) with respect to the indicated delay of the laser pulse and with a 150W Xe pulsed lamp. The kinetics were obtained with a photomultiplier detector and the pulsed Xe lamp. Optically-matched solutions (optical density of 0.5) were used in 1 cm septum-stoppered quartz cuvettes (standard cross-beam geometry) under nitrogen- and oxygen-saturated conditions.

**Attenuated total reflectance-Fourier transform infrared spectroscopy.** The ATR-FTIR spectra were collected on a Bruker alpha II FTIR spectrometer in the 4000−400 cm^−1^ range. A few drops were added of a concentrated solution/dispersion, and the solvent was completely dried.

**Transmission electron microscopy.** Transmission electron microscopy (TEM) was performed on a HITACHI HT7800 microscope with a filament of LaB_6_ operated at 100keV.

**Dynamic Light Scattering and Zeta Potential.** Dynamic Light Scattering (DLS) and Zeta Potential (ζ-potential) measurements of NC [111 nM] and NC@ZnPc (1:40 NC/ZnPc molar ratio) dispersions in toluene were performed with a Zetasizer Ultra instrument (Malvern, UK).

**Calculation of energy transfer rate constants (*k*_ET_)**

Energy transfer rate constants (s^-1^) were calculated according to the following equation:

$k_{\mathrm{ET}}= \frac{1}{\tau_{NC@ZnPc}-\frac{1}{\tau_{\mathrm{NC}}}}$ (S1)

where $k_{ET}$ is the rate constant for the energy transfer process calculated independently for the lifetime components (X, STX, and DTX), $\tau_{NC@ZnPc}$ , and $\tau_{\mathrm{NC}}$ are the lifetimes of each component ($\tau_{X}$, $\tau_{\mathrm{STX}}$, and $\tau_{\mathrm{DTX}}$) for the NC in the presence of different concentrations of ZnPc and the pristine NC, respectively.

**Calculation of energy transfer time constants (τ_ET_)**

Energy transfer time constants (ns) were calculated according to the following equation:

$\tau_{ET}= \frac{1}{k_{ET}}$ (S2)

**Calculation of energy transfer quantum yields (*Φ*_ET_)**

Energy transfer quantum yields (%) were calculated according to the following equation:

$\Phi_{ET}=\left( 1-\frac{\tau_{NC@ZnPc}}{\tau_{NC}} \right)*100$ (S3)

**Estimation of theoretical energy transfer rate constants (*k*_ET_)_t_**

Estimation of theoretical energy transfer rate constant assuming a purely Förster-based model was done by applying the following formalism:

$k_{(ET)t}= \frac{1}{\tau_{\mathrm{NC}}}{(\frac{R_{0}}{r_{\mathrm{DA}}})}^{6}$ (S4)

where $\tau_{\mathrm{NC}}$ is the lifetime of the STX component in absence of quencher (2.12 ns, Table S1), $R_{0}$ is the Förster distance at which the efficiency of energy transfer between the NC and ZnPc is 50% and depends on several factors, including the spectral overlap constant between the NC emission and ZnPc absorption (${J(\varepsilon}_{A})$ = 3.08 10^13^ nm^4^M^-1^cm^-1^) calculated from a|e 1.2 UV-Vis-IR spectral software from FluorTools, NC quantum yield ($\Phi_{\mathrm{NC}}$ = 0.47), toluene refractive index ($n$ = 1.497) and the relative orientation of the transition dipoles ($\kappa$ = 2/3 for random dipoles usually employed for inorganic semiconductor and organic dyes). The $r_{\mathrm{DA}}$ is the center-to-center distance between NC and ZnPc, calculated by measuring the size from TEM images (3.25 nm for NC radius) and by measuring the molecule radius in ChemDraw software, version 22.2.0.3300 (0.83 nm for ZnPc radius).

$R_{0}^{6}=\frac{\Phi_{D}\kappa^{2}9000(ln(10))}{{128\pi}^{5}{Nn}^{4}}{J(\varepsilon}_{A})$ (S5)

Thus, accounting for $\tau_{\mathrm{NC}}$ = 2.12 ns (STX), $R_{0}$ = 3.45 nm and $r_{\mathrm{DA}}$ = 4.08 nm, the theoretical energy transfer rate constant is estimated to be $k_{(ET)t}$ = 0.17 10^9^ s^-1^.


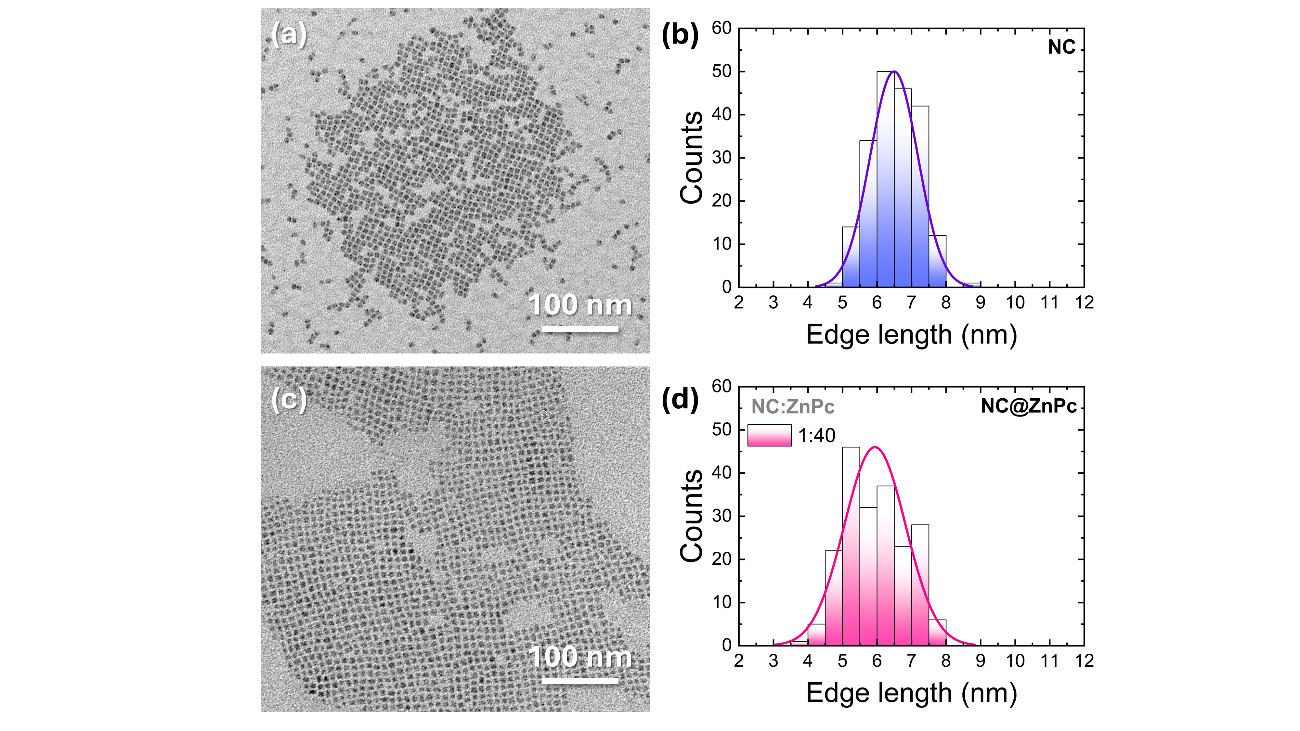


**Figure S1.** (a) Representative TEM images of NCs and (c) NC@ZnPc (ratio NC: ZnPc of 1:40) nanohybrids along with their corresponding size distributions (b) and (d), respectively.

**Aggregation-disaggregation phenomena**

The typical absorption spectrum of ZnPc registered in polar solvents such as ethanol at low concentration (0.8 µM) presents a narrow band at 677 nm (Q-band) and a shoulder in the range 600-650 nm.^[19]^ The absorption features of ZnPc in toluene, recorded at the higher concentration used to prepare the NC@ZnPc nanohybrid (10 µM), displayed a Q-band with two equally intense peaks at 671 nm and 684 nm resulting from the extended π-conjugation,^[15]^ and a shoulder at 637 nm (**Figure 1a**, blue spectrum) that could be assigned to the dye-aggregation. Similar absorption spectrum was reported for the ZnPc at high concentration (50 µM) in solvent mixture of *t*-butanol:acetonitrile as the typical absorption feature of the ZnPc aggregate.^[19]^

Additional measurements were performed to analyze the aggregation-disaggregation phenomenon of dye in the nanohybrid: i) ZnPc absorption spectra (0-10 µM) were compared to those of the dyes in the nanohybrid (Figure S3a,c); ii) the ZnPc molar absorption coefficient (at 680 nm) was calculated showing an increase in the nanohybrids from 1.08x10^5^ M^-1^cm^-1^ to 1.77x10^5^ M^-1^cm^-1^ (Figure S3b,d); iii) ZnPc absorption spectra were recorded at increasing concentration of NCs (0-30 nM), exhibiting a reduction in the aggregate band with the concomitant bathochromic shift of the highest-energy Q-band (Figure S4). All these observations agree well with the different environment of the ZnPc in the NC surface and the dye disaggregation attributed to the interdigitation of the organic ligands, oleic acid and oleylamine, within the ZnPc dye aggregates. It is noteworthy that oleic acid (OA) together with chenodeoxycholic acid (CDCA) was added as co-adsorbate to reduce the aggregation of ZnPc in the dye self-assembled monolayer (SAM) deposition on TiO_2_. Although OA had an evident dye-disaggregation effect, it was lower than the observed for CDCA explained by OA lipidic structure in the SAM formation that led to a higher surface density.^[19]^


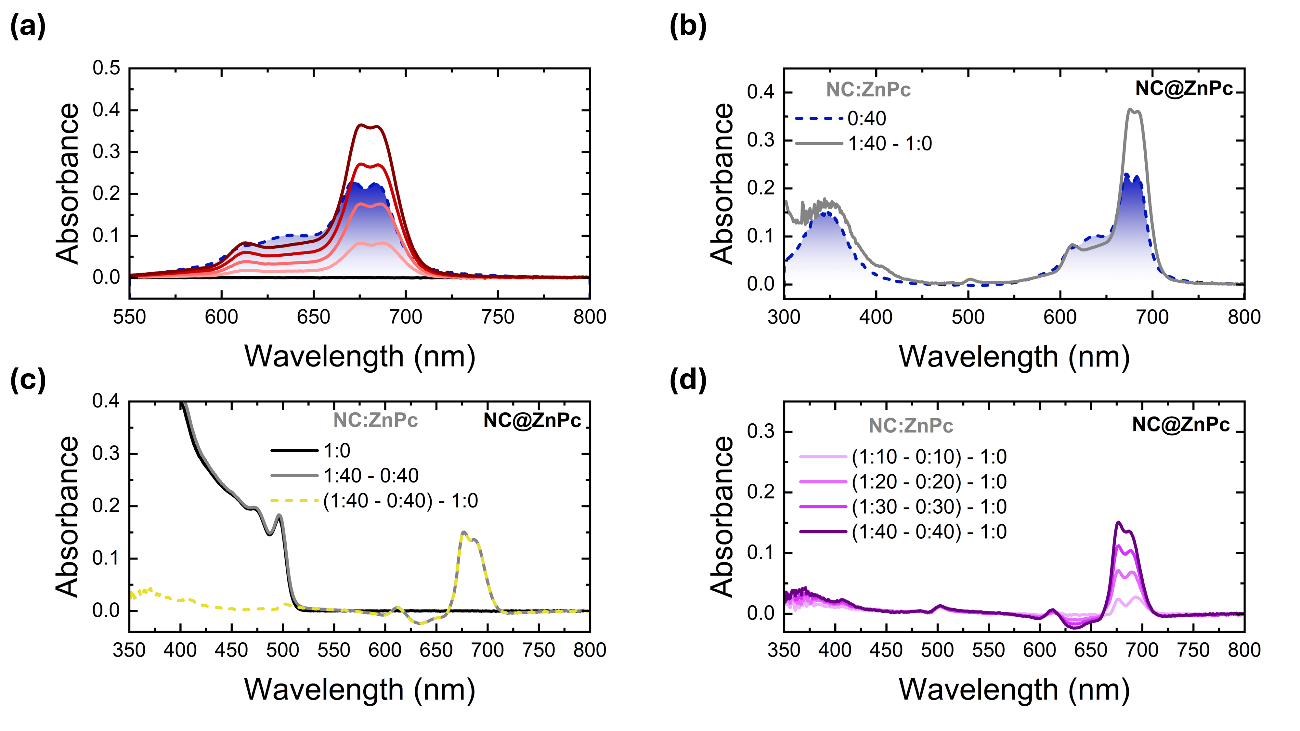


**Figure S2.** (a) Steady-state absorption spectra of NCs at increasing concentrations of ZnPc [0-10 μM] (zoom-in from Figure 1a). The blue curve is ZnPc [10 μM] reference. (b) Comparison of the steady-state absorption spectra of ZnPc control [10 μM] and subtracted ZnPc from NC:ZnPc [0.25 μM: 10 μM] and NC [0.25 μM]. (c) Comparison of the steady-state absorption spectra of NCs, subtracted NCs, and electronic interaction for a NC:ZnPc ratio of 1:40. (d) The same for (c) but with all concentrations.


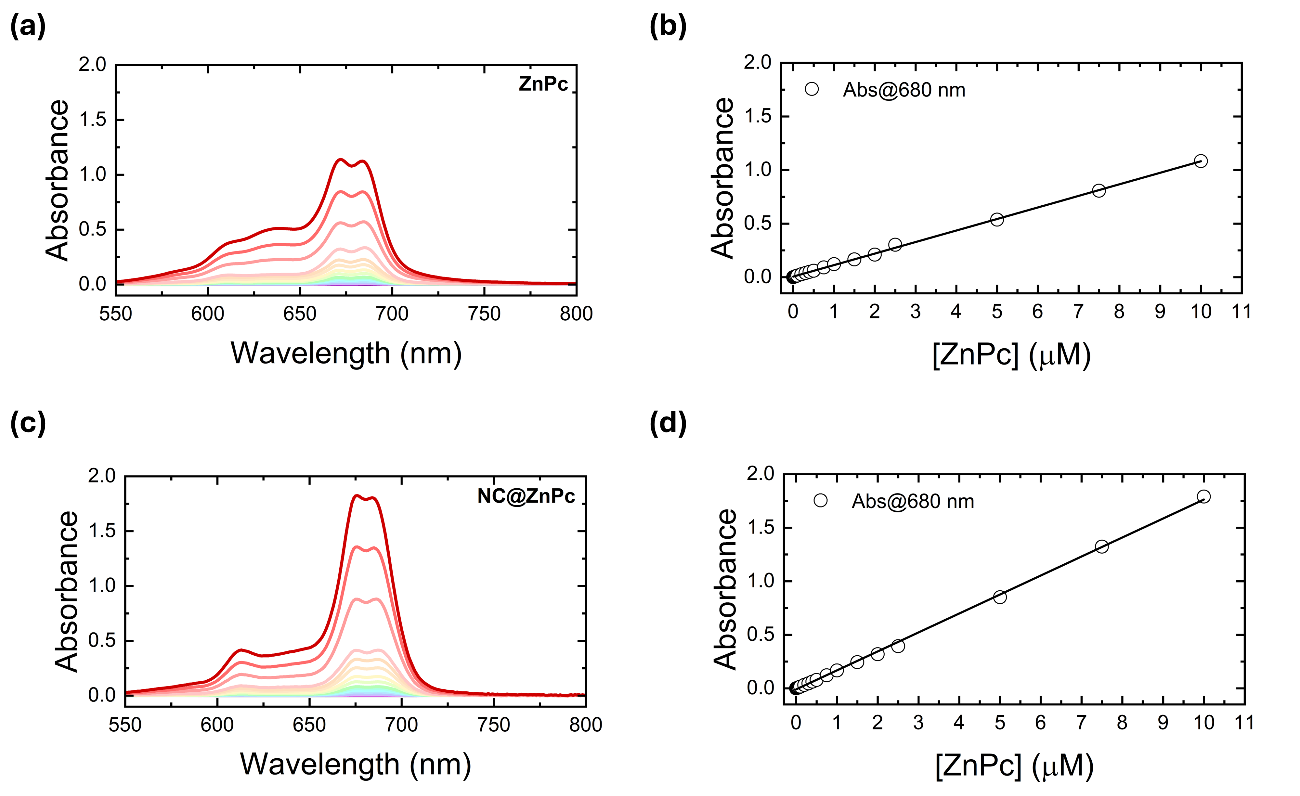


**Figure S3.** (a) Steady-state absorption spectra of ZnPc [0-10 μM] and (c) NC:ZnPc [0.25 μM:0-10 μM] along with their corresponding Lambert-Beer relationships in (b) and (d), respectively.


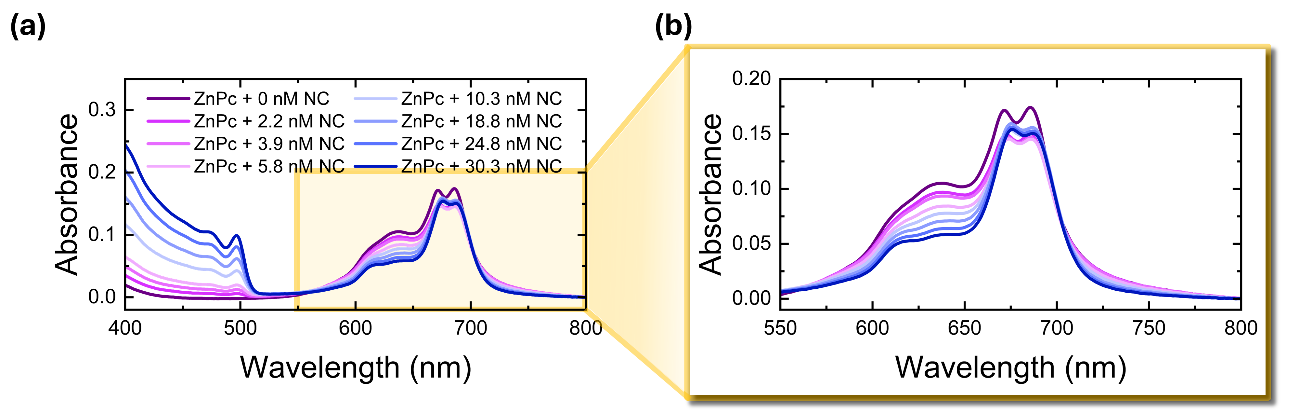


**Figure S4.** (a) Steady-state absorption spectra of ZnPc at increasing concentrations [0-30.3 nM] of NCs. (b) Zoom-in in the 550-800 nm range.


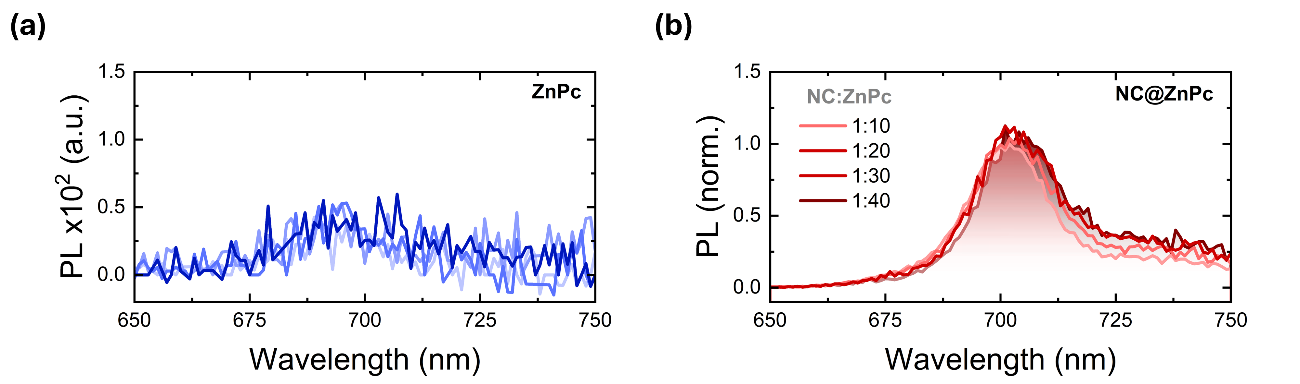


**Figure S5.** (a) Steady-state PL spectra of ZnPc [2-10 μM] and (b) normalized PL spectra of NC@ZnPc nanohybrid upon 460 nm photoexcitation at increasing concentrations of ZnPc [2-10 μM].


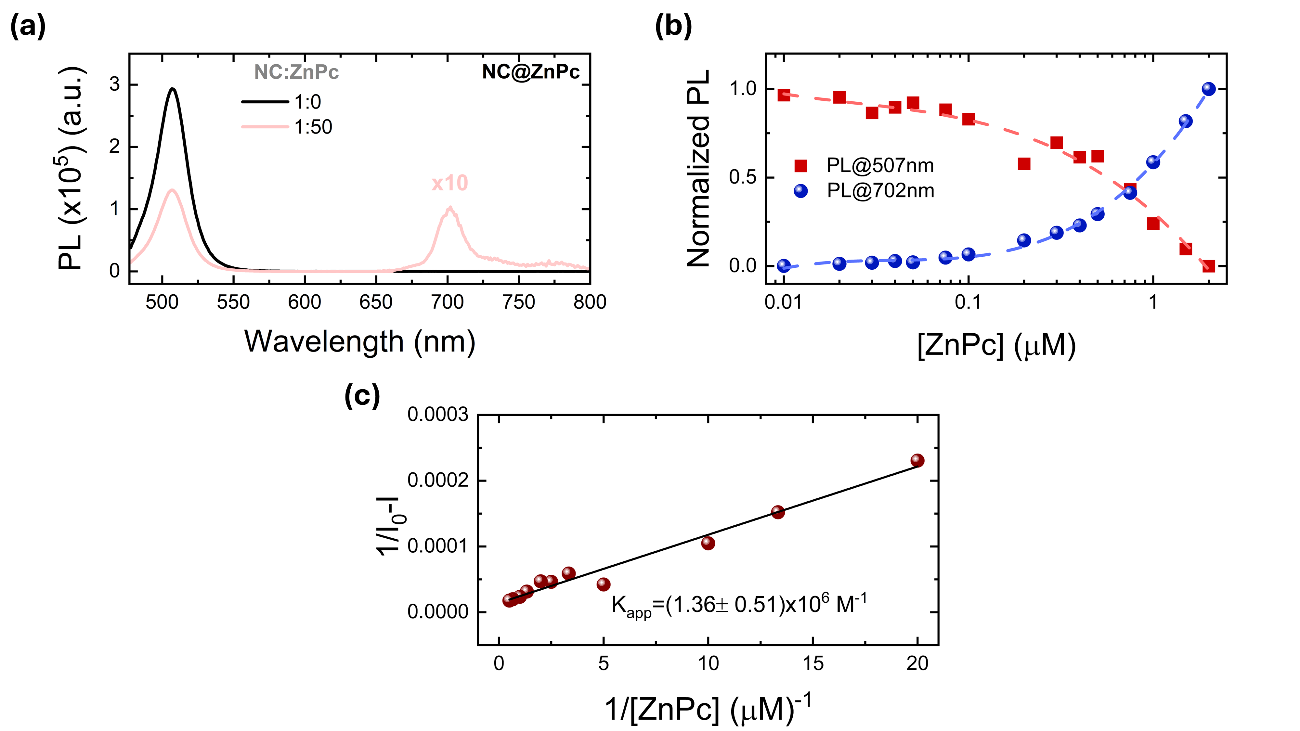


**Figure S6.** (a) Steady-state PL spectra of pristine NCs [0.04 μM] and NC@ZnPc (1:50) nanohybrid under 460 nm photoexcitation. (b) PL evolution at the NC maximum (507 nm) and ZnPc maximum (702 nm) at increasing concentrations of ZnPc [0-2 μM]. (c) Double-reciprocal analysis of the NC PL quenching.


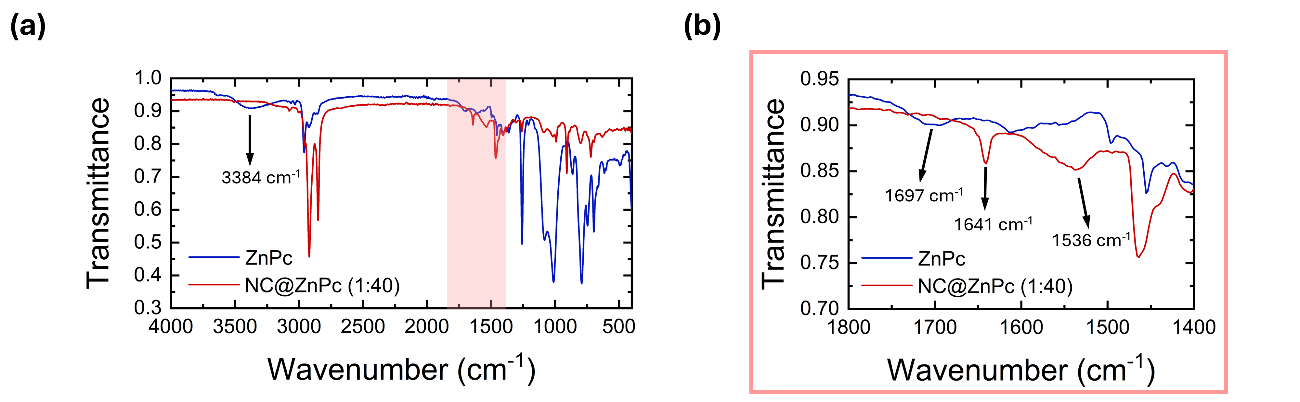


**Figure S7.** (a) ATR-FTIR spectra of the ZnPc and NC@ZnPc (1:40) nanohybrid. (b) Enlarged region of (a).


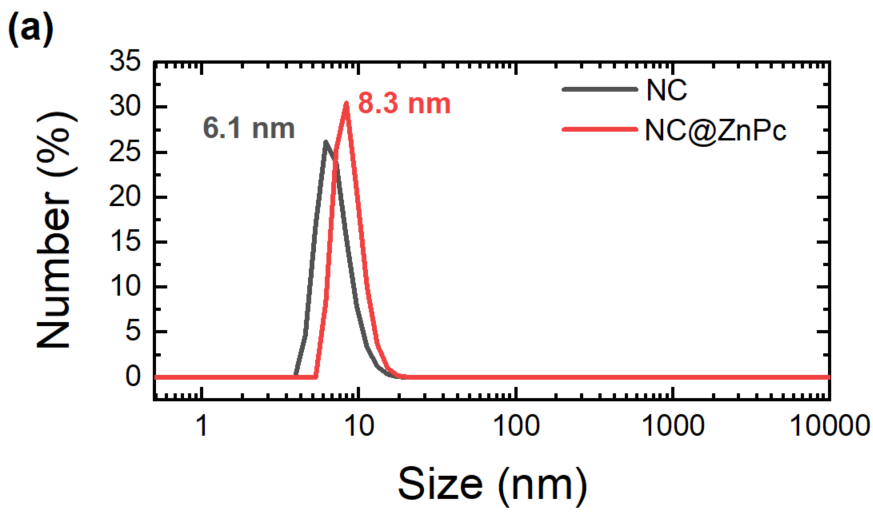

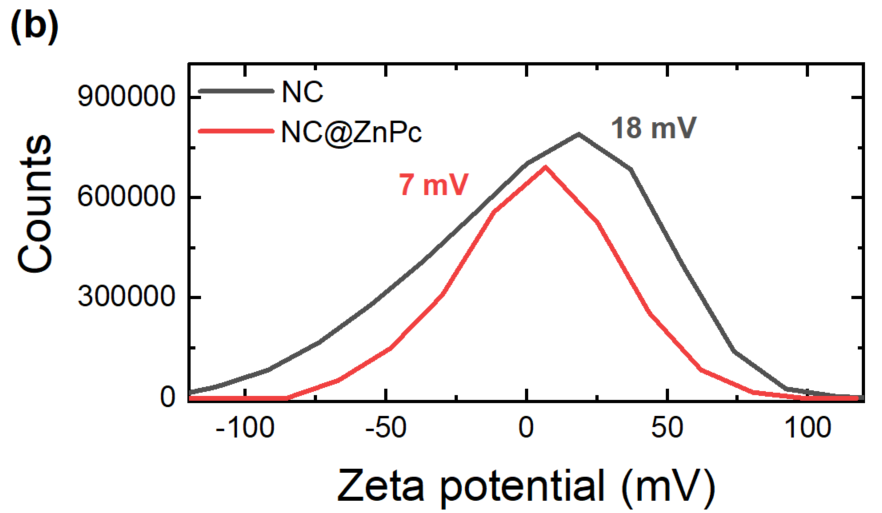


**Figure S8.** Size distribution from dynamic light scattering (a) and Zeta potential (b) for NC and NC@ZnPc (1:40) nanohybrid.


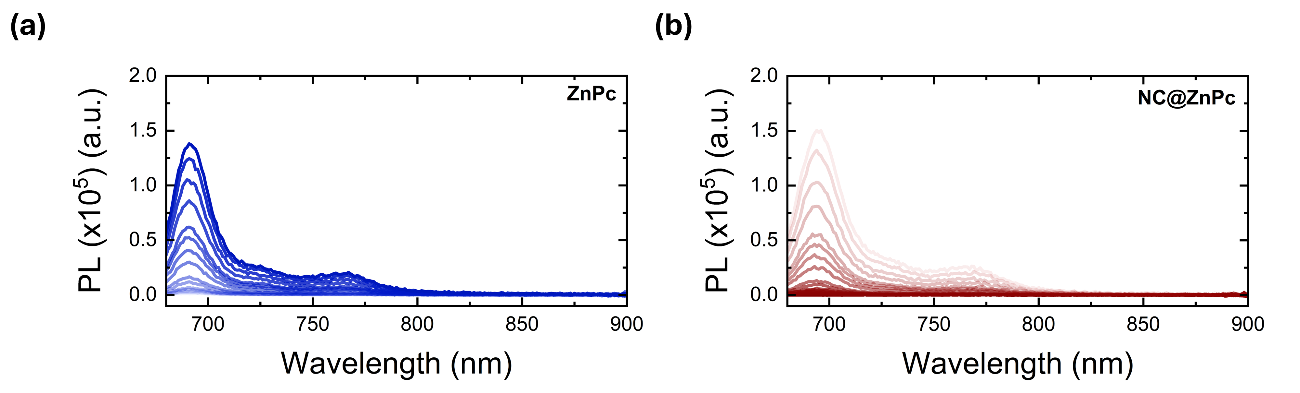


**Figure S9.** (a) Steady-state PL spectra of ZnPc [0-2 μM] and (b) NC@ZnPc (1:50) nanohybrid under 670 nm photoexcitation at increasing concentrations of ZnPc [0-2 μM].


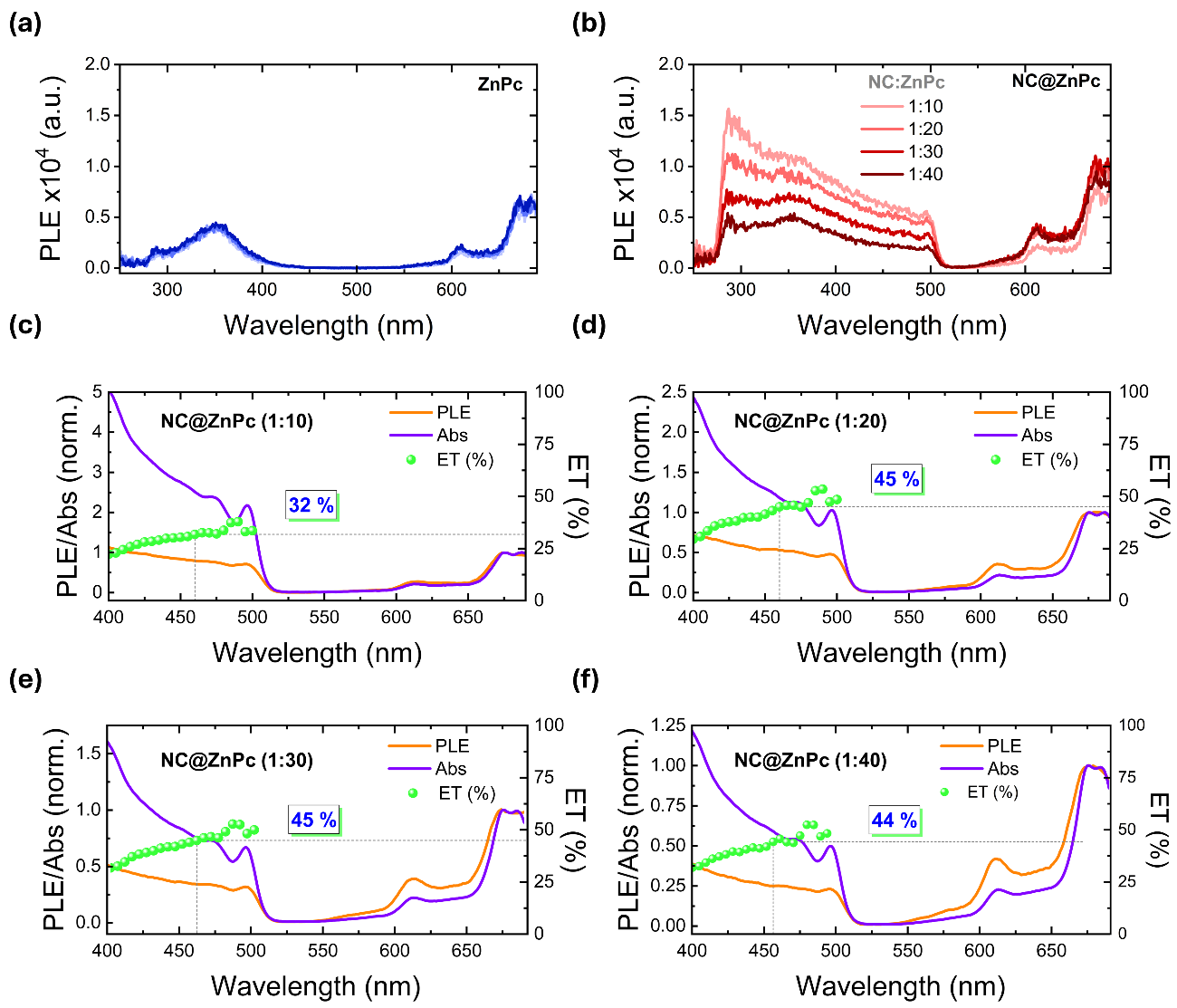


**Figure S10.** (a) Steady-state PLE spectra of ZnPc [2.5-10 μM] and (b) NCs at increasing concentrations of ZnPc [0-10 μM] monitored at the fluorescence wavelength of 702 nm. Comparison of the PLE and absorption spectra and corresponding energy transfer estimation for NC@ZnPc nanohybrids for NC:ZnPc ratios of (c) 1:10, (d) 1:20, (e) 1:30 and (f) 1:40.


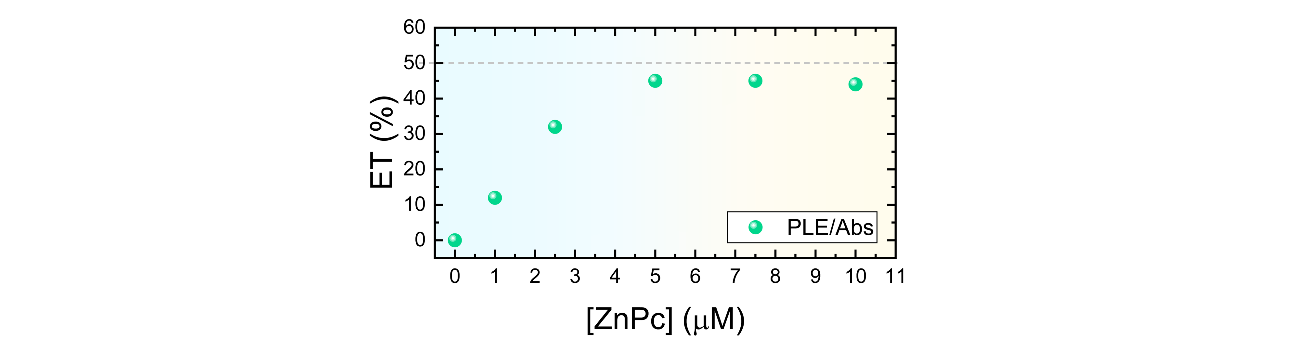


**Figure S11.** Energy transfer efficiency from NCs to surface-anchored ZnPc at increasing ZnPc concentration [0-10 μM] calculated from Figure S9.


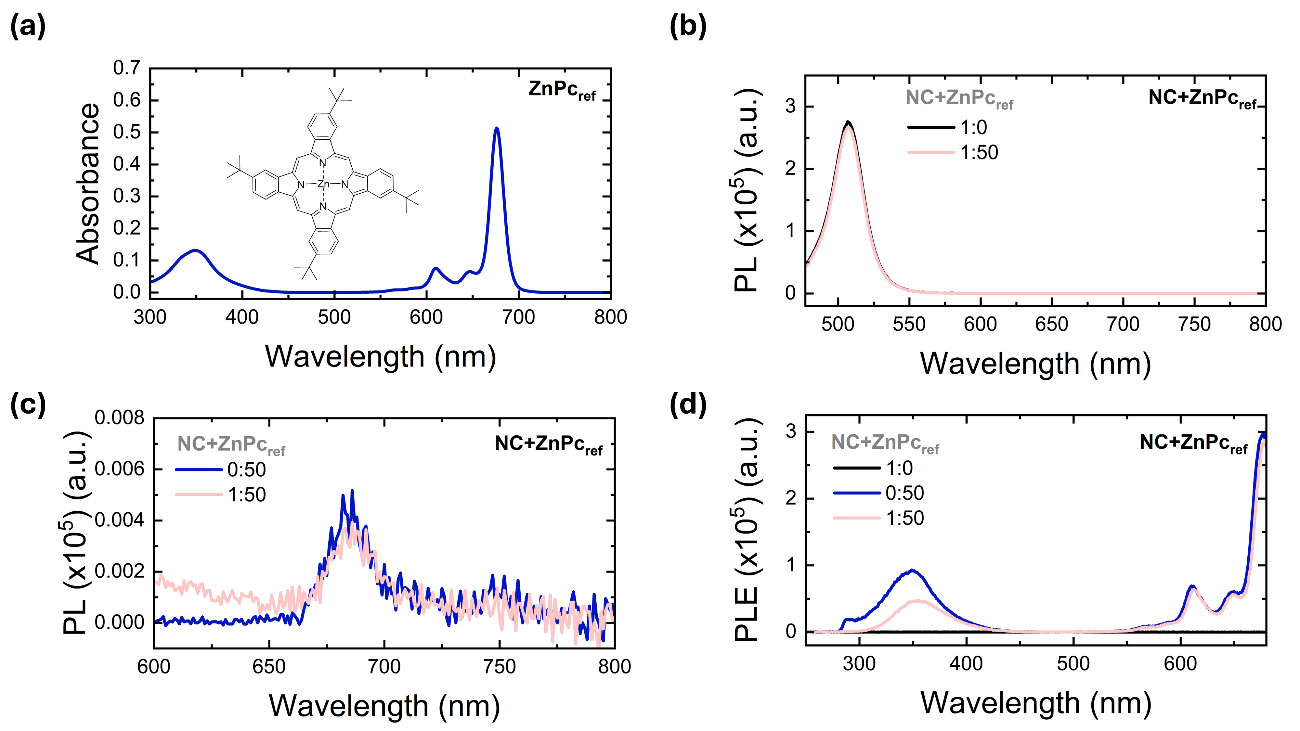


**Figure S12.** (a) Steady-state absorption spectrum of the ZnPc_ref_ [2 µM]. (b) Steady-state PL spectra of NCs with and without x50 of ZnPc_ref_ under 460 nm photoexcitation. (c) Steady-state PL spectra of NCs with and without x50 of ZnPc_ref_ under 460 nm photoexcitation. (d) Steady-state PLE spectra of NCs, ZnPc_ref_ and mixture monitored at 702 nm.


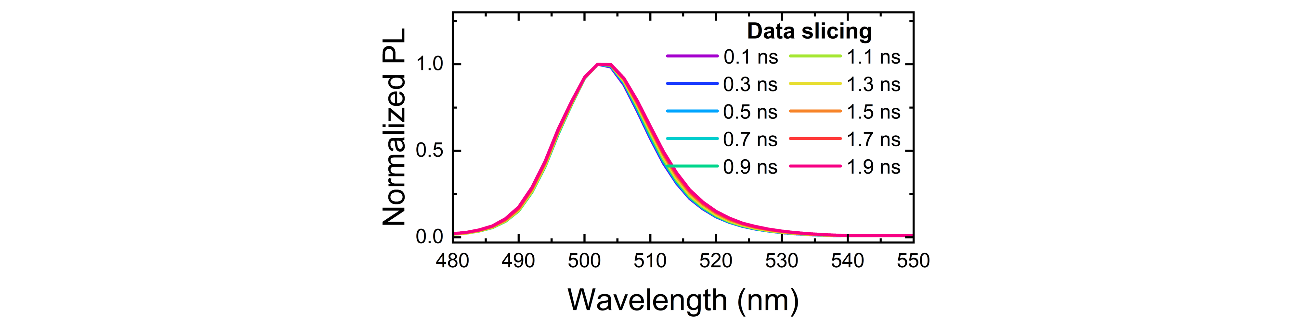


**Figure S13.** Time-resolved emission spectra (TRES) map of NCs under 460 nm pulsed laser photoexcitation and 507 nm detection. Data slicing at indicated delay times.


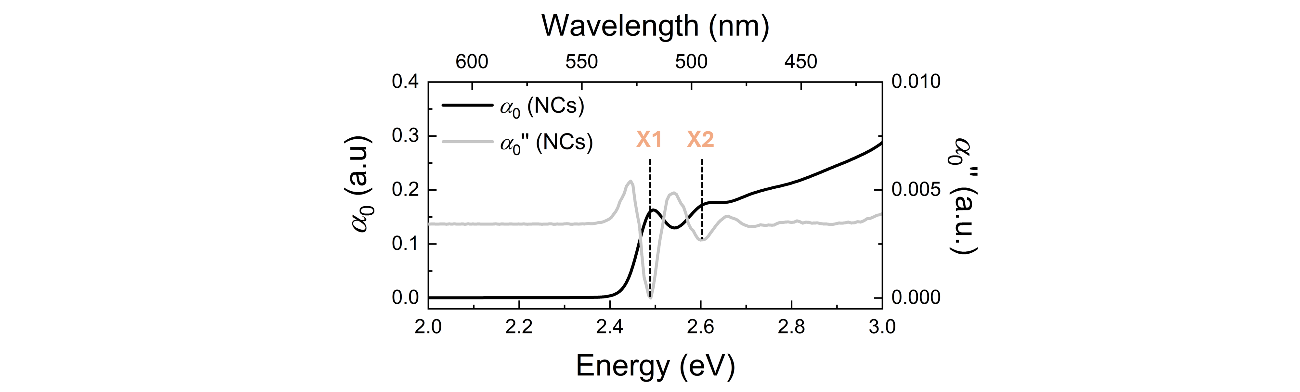


**Figure S14.** Steady-state absorption spectrum of pristine NCs (black curve) and its second derivative (gray curve).

**Pump energy-dependent studies**

It is worth noting that high excitation fluences were used to track the sensitized ZnPc features. The fluence-dependence fs-transient absorption probed at XB after 200 ps time delay, represented in Figure S15, clearly showed a deviation from linearity in the first regime of fluence density as expected due to the high photon fluences used in the whole experimental range (1.8 x 10^15^ – 1.5 x 10^16^ photons/cm^2^·s). Therefore, we were unable to estimate the exciton occupancy per nanocrystal precisely from this experiment. Nevertheless, based on the cross-section values for CsPbBr_3_ NC (ca. 1 10^-14^ cm^2^ at 450 nm) from the literature,^[43f]^ we have estimated the exciton occupancy per nanocrystal <*N*> for the lowest fluence used (0.76 μJ/cm^2^), as 18. Thus, confirming the multi-exciton formation regime for the complete range.

A fluence-dependent TAS study in the strong excitation regime (0.15-1.25 μJ) was conducted to assess the properties of multiexcitonic states (Figure S16b). A fast component in the XB feature (inverted) emerges in the early time window as the pump fluence is increased. This fast component is a typical signature of multiexcitons generated in QDs and NCs by high excitation fluences as previously reported in other studies.^[26, 36b]^ This multiexcitonic state usually decays via nonradiative Auger-assisted processes, where the energy of the electron-hole pair is transferred to a third nearby charge carrier (electron/hole). The fast-decaying component can be extracted by performing a subtraction between two kinetic traces corresponding to 1.25 and 0.15 μJ after normalizing them to the absorption tail at 200 ps, where multiexciton components have completely decayed and the exciton component is only present. The subtracted kinetic trace was fitted to a monoexponential decay with a time constant of 35 ps (Figure 16c), which is in the time range of biexciton components already reported.^[26, 31, 37, 43d, 43e]^ This biexciton time constants in perovskite NCs is even shorter than those found in traditional CdSe and PbSe QDs of similar sizes,^[26]^ limiting the efficiency of devices relying on multiexciton generation. Figure S16f shows a schematic illustration of the exciton and multiexciton behavior under low and high excitation fluences, respectively.


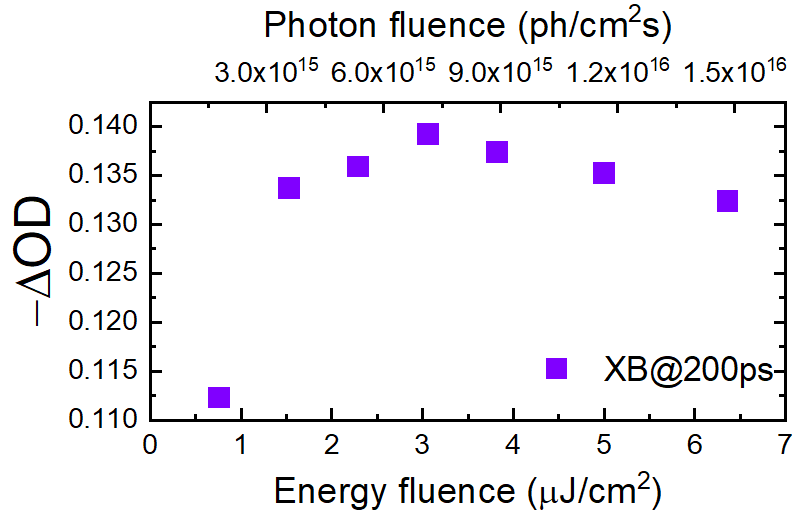


**Figure S15:** Fluence-dependence fs-transient absorption probed at XB (498 nm) after 200 ps time delay.


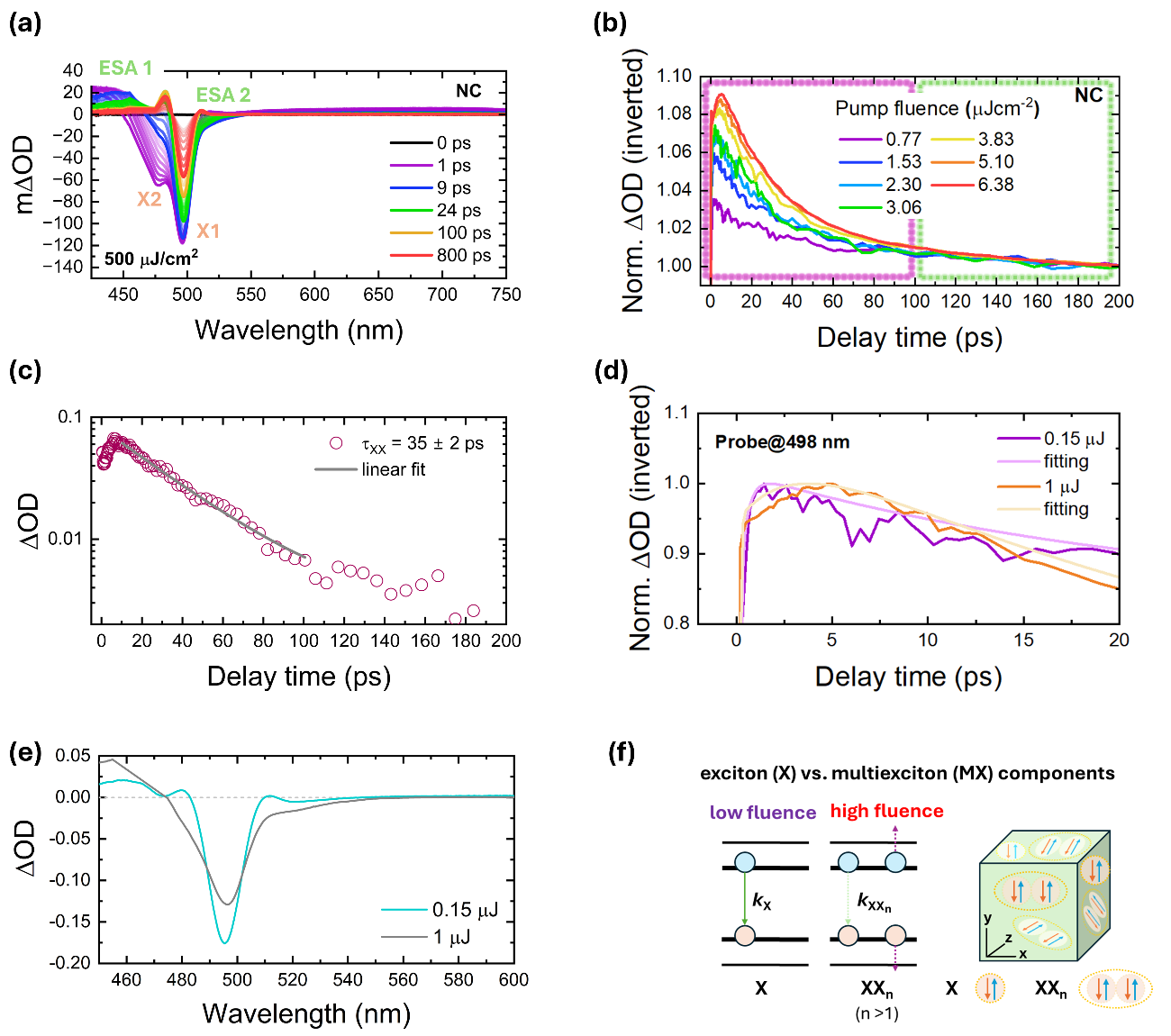


**Figure S16.** (a) fs-TA spectra of pristine NCs at indicated delay times under pump energy of 1 μJ. (b) Inverted and normalized TA kinetics at 200 ps of the pristine NCs at 498 nm (XB) under indicated pump energies. (c) Kinetic trace obtained by subtraction of the kinetic trace at high and low pump energies and corresponding fitting. (d) Inverted and normalized XB kinetics under low and high pump energy. (e) SADS for the (MX) component in the NCs under 0.15 and 1 μJ pump energy. (f) Representation of the exciton and multiexciton deactivation generated under low and high fluences, respectively.


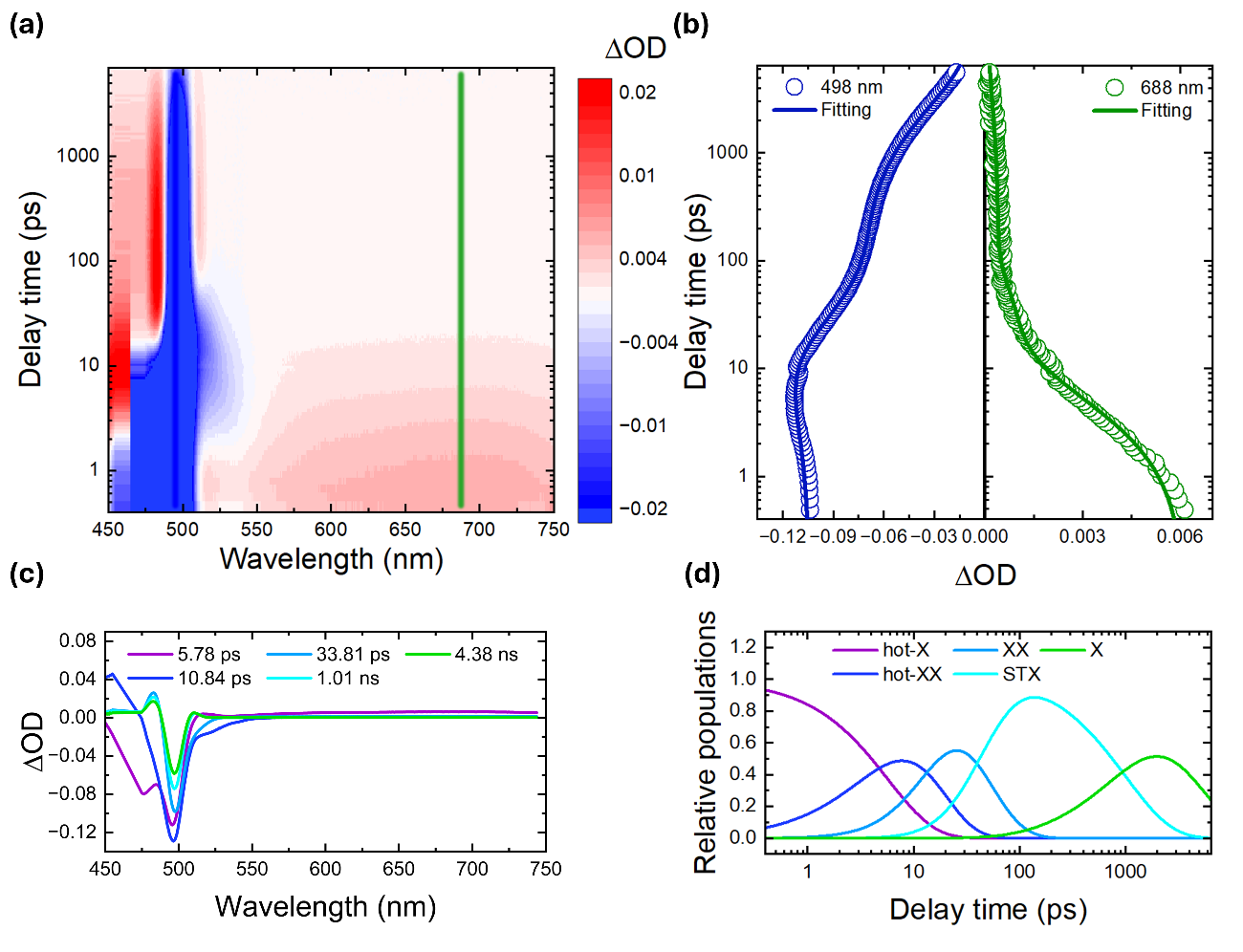


**Figure S17**. fs-TAS of standalone NCs. (a) fs-2D pseudo-color plot of raw data. (b) Representative decay traces and fitting at 498 and 688 nm. (c) Species-associated differential spectra (SADS) obtained by target analysis – see figure legend for lifetime assignment. (d) Corresponding relative populations – see figure legend for species assignment.


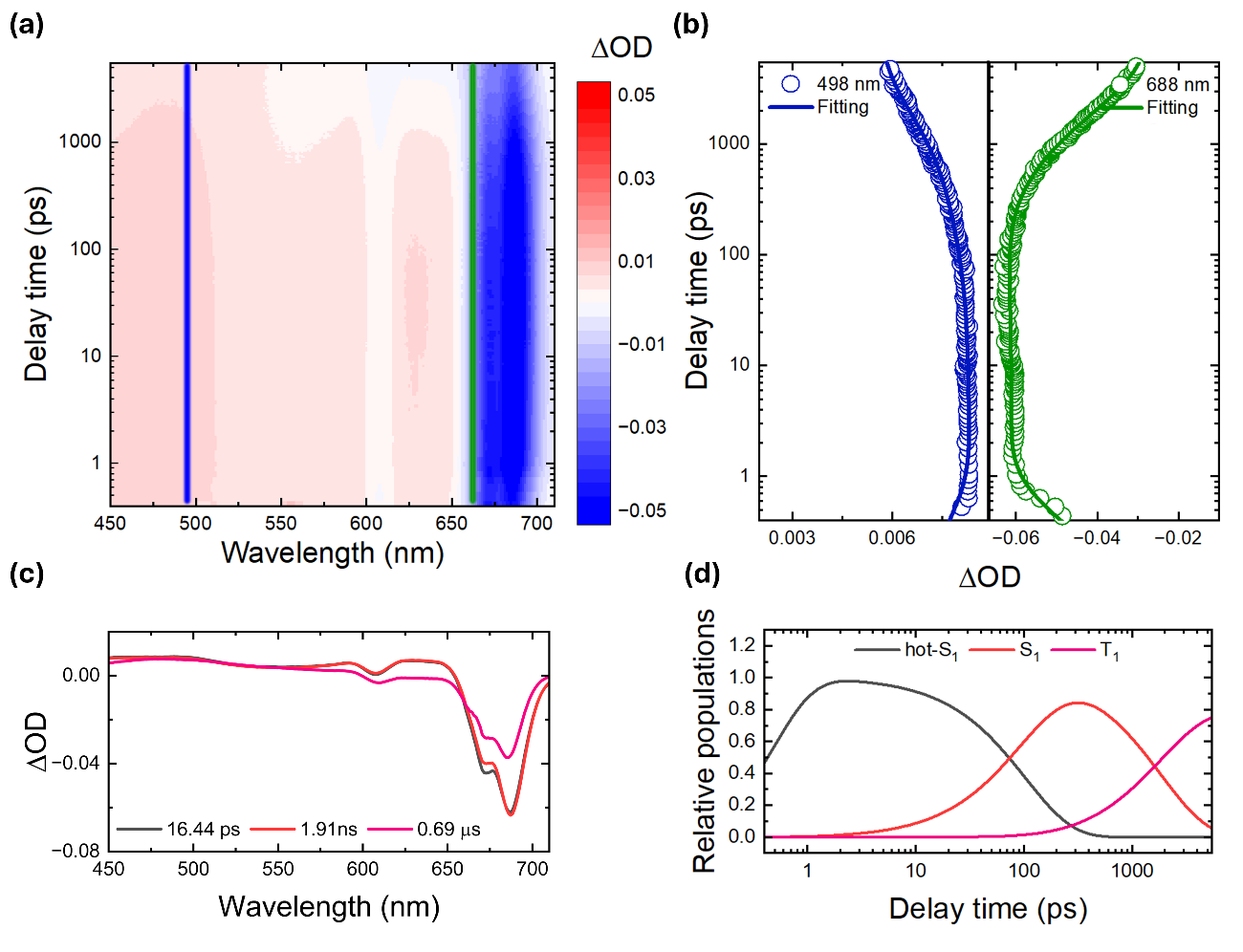


**Figure S18.** fs-TAS of ZnPc. (a) fs-2D pseudo-color plot of raw data. (b) Representative decay traces and fitting at 498 and 688 nm. (c) Species-associated differential spectra (SADS) obtained by target analysis – see figure legend for lifetime assignment. (d) Corresponding relative populations – see figure legend for species assignment.


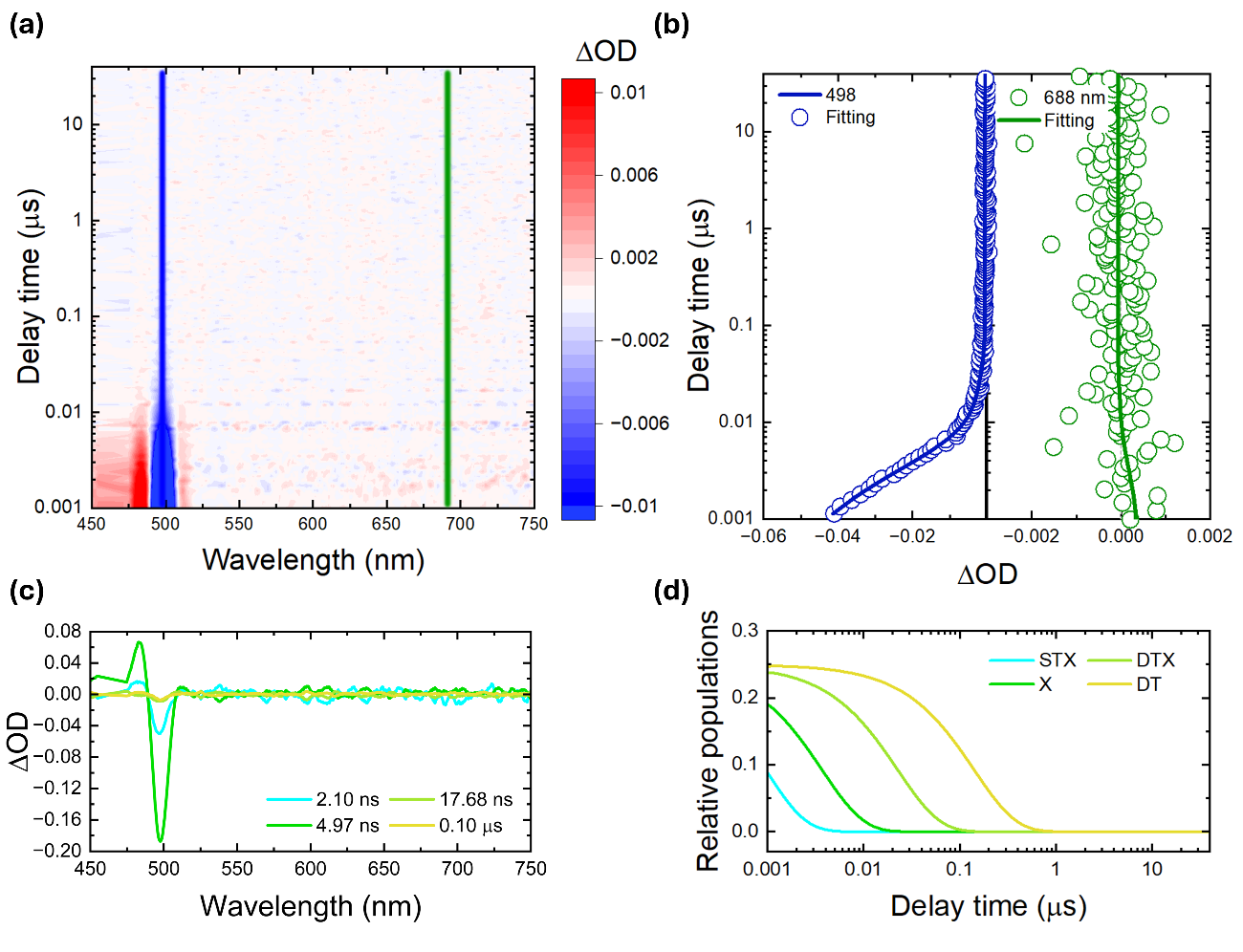


**Figure S19.** ns-TAS of standalone NCs. (a) fs-2D pseudo-color plot of raw data. (b) Representative decay traces and fitting at 498 and 688 nm. (c) Species-associated differential spectra (SADS) obtained by target analysis – see figure legend for lifetime assignment. (d) Corresponding relative populations – see figure legend for species assignment.


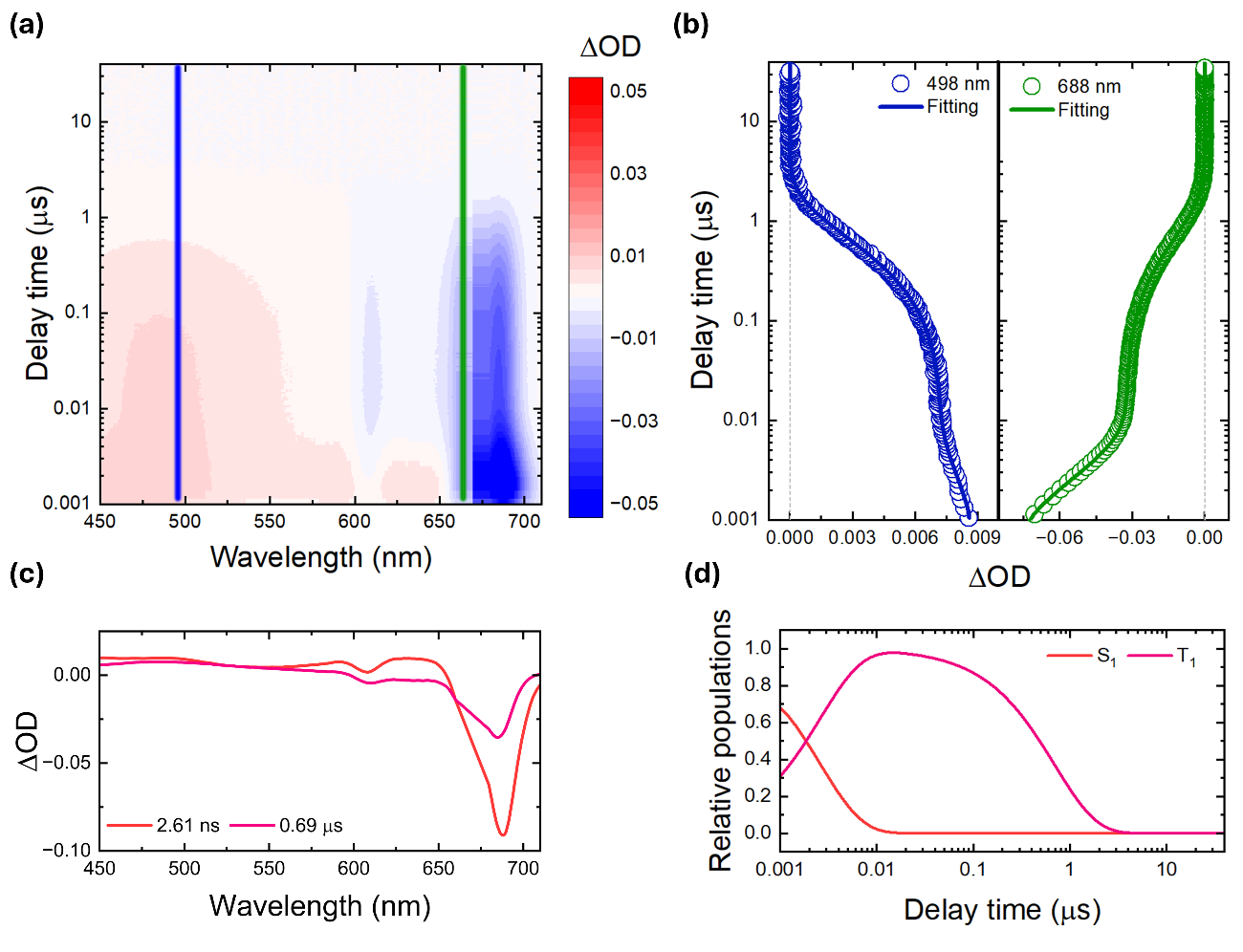


**Figure S20.** ns-TAS of ZnPc. (a) fs-2D pseudo-color plot of raw data. (b) Representative decay traces and fitting at 498 and 688 nm. (c) Species-associated differential spectra (SADS) obtained by target analysis – see figure legend for lifetime assignment. (d) Corresponding relative populations – see figure legend for species assignment.


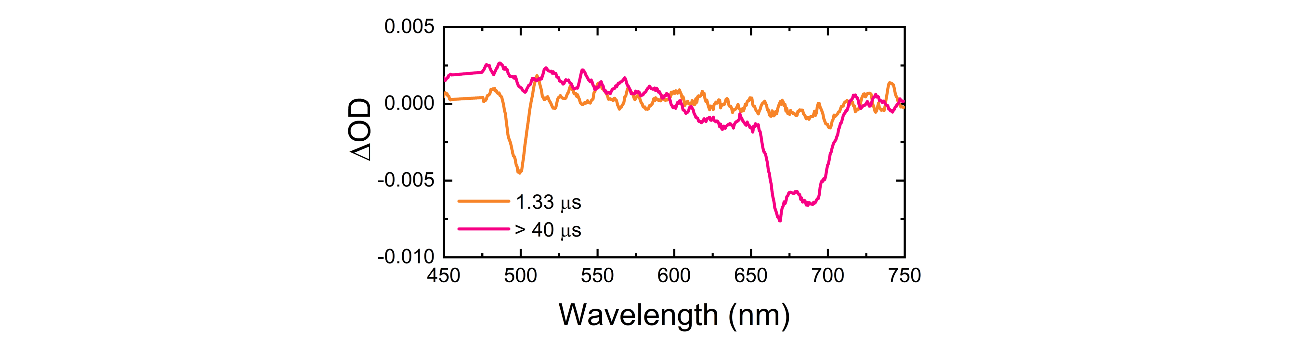


**Figure S21.** SADS of the new perovskite trap state (DT’) and ZnPc triplet excited state with time constants of 1.33 and >40 μs, respectively obtained by target analysis.

**
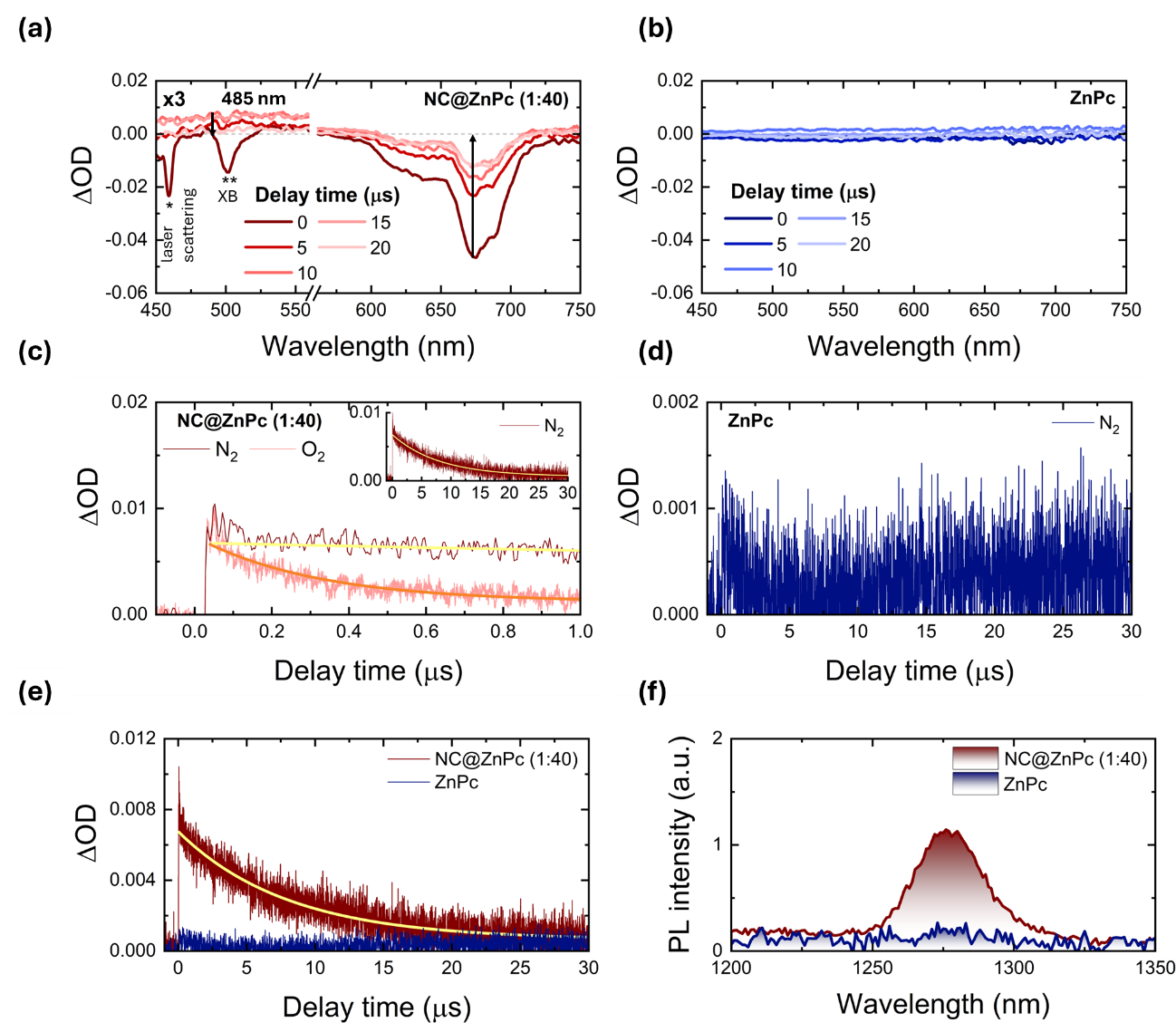
**

**Figure S22.** µs-TA spectra at indicated delay times for (a) NC@ZnPc (1:40) nanohybrid and (b) ZnPc control under 460 nm pump excitation under nitrogen conditions. (c) Kinetic traces at 485 nm for the NC@ZnPc (1:40) nanohybrid under nitrogen and oxygen conditions. Inset: complete trace under nitrogen conditions. (d) Kinetic trace at 505 nm for the ZnPc control. (e) Comparative of kinetic traces for NC@ZnPc (1:40) nanohybrid and ZnPc control. (f) Singlet oxygen PL spectra for NC@ZnPc (1:40) nanohybrid and ZnPc control under 460 nm excitation.

**
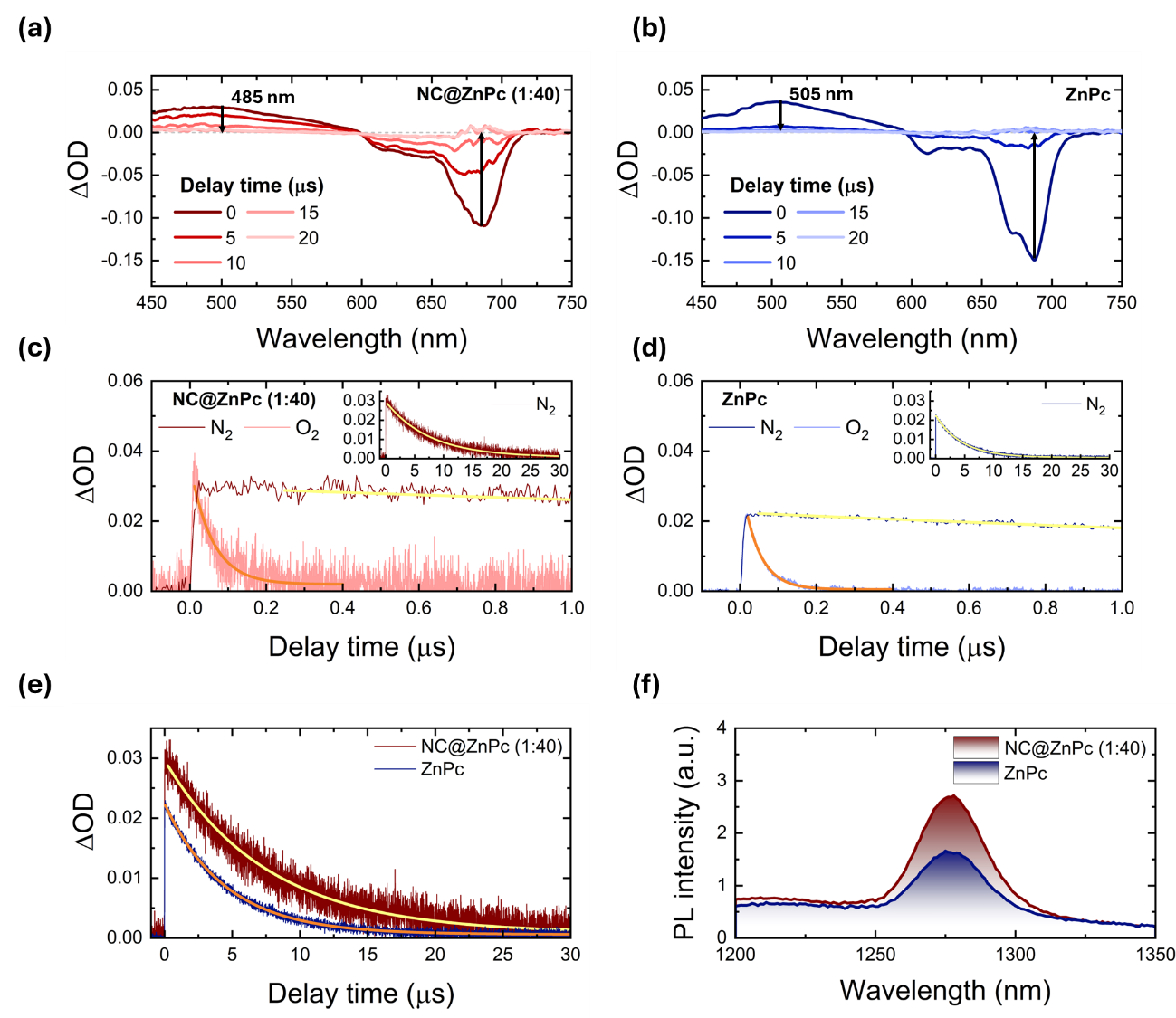
**

**Figure S23.** µs-TA spectra at indicated delay times for (a) NC@ZnPc (1:40) nanohybrid and (b) ZnPc control under 620 nm pump excitation under nitrogen conditions. (c) Kinetic traces at 485 nm for the NC@ZnPc (1:40) nanohybrid under nitrogen and oxygen conditions. Inset: complete trace under nitrogen conditions. (d) Kinetic trace at 505 nm for the ZnPc control under nitrogen and oxygen conditions. Inset: complete trace under nitrogen conditions. (e) Comparative of kinetic traces for NC@ZnPc (1:40) nanohybrid and ZnPc control. (f) Singlet oxygen PL spectra for NC@ZnPc (1:40) nanohybrid and ZnPc control under 620 nm excitation.


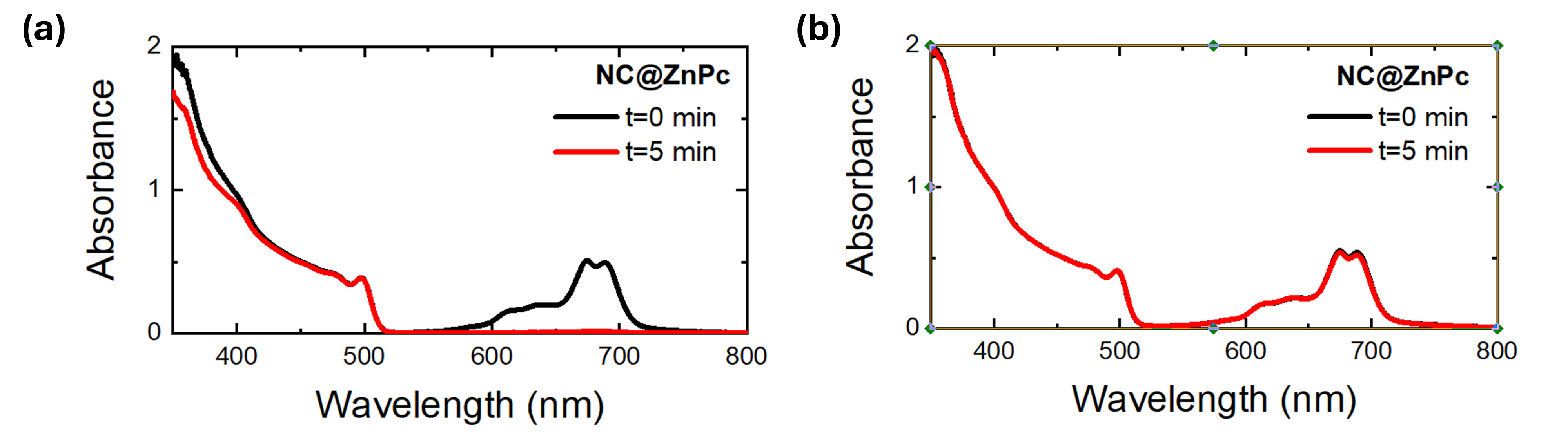


**Figure S24.** Absorption spectra of NC@ZnPc nanohybrid (NC:ZnPc ratio of 1:40) before and after continuous light excitation at (a) 445-465 nm and (b) 630-650 nm.


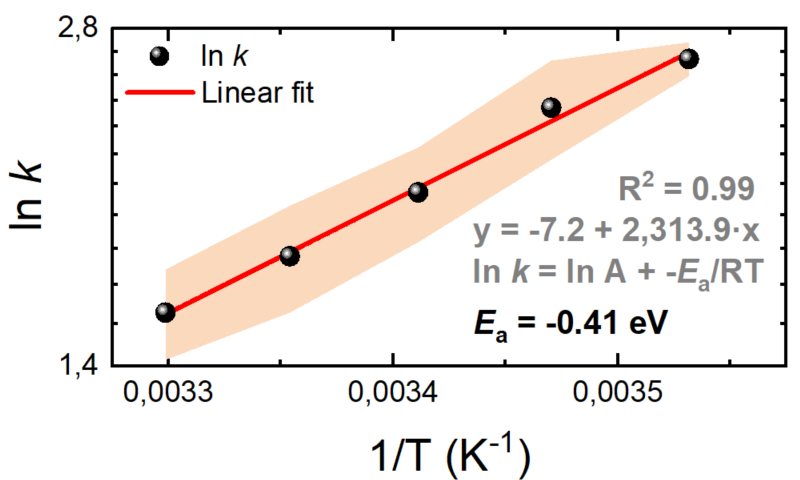


**Figure S25.** Arrhenius plot for the ZnPc (S_1_) formation rate constant at variable temperatures.


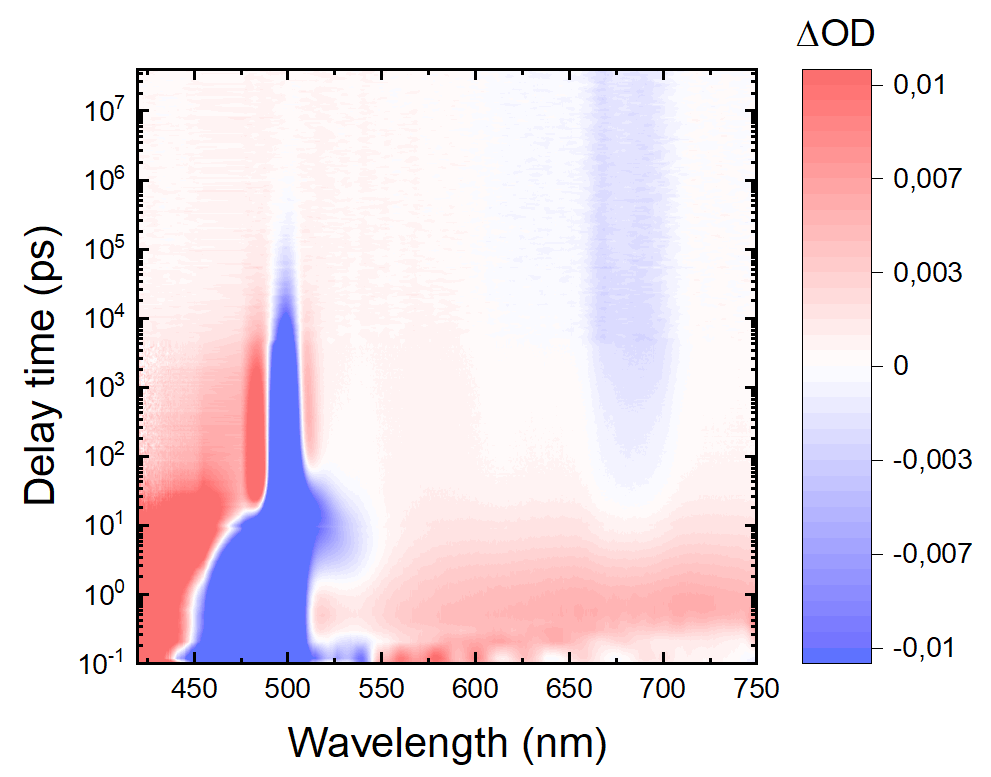


**Figure S26:** Combination of all photophysical processes (from 0.5 ps-40 µs) involved in the system represented for the NC@ZnPc nanohybrid in the 400-750 nm range.

**Table S1.** Time constants derived from TCSPC measurements.

| **TCSPC** | **λ_em_ (nm)** | **τ_STX_**  **±sd (ns)** | **B_STX_ (%)** | **τ_X_**  **±sd (ns)** | **B_X_ (%)** | **τ_DTX_ ±sd (ns)** | **B_DTX_ (%)** | **τ_R_**  **±sd (ns)** | **τ_S1_**  **±sd (ns)** | **B_S1_ (%)** | **τ_S1’_**  **±sd (ns)** | **B_S1’_ (%)** | ***χ*^2^** |
| --- | --- | --- | --- | --- | --- | --- | --- | --- | --- | --- | --- | --- | --- |
| NC | 507 | 2.12  ±0.09 | 16 | 4.97  ±0.06 | 76 | 17.68  ±0.53 | 8 |  |  |  |  |  | 1.19 |
| NC  +10eq ZnPc |  | 0.57  ±0.01 | 30 | 2.90  ±0.03 | 57 | 10.36  ±0.18 | 13 |  |  |  |  |  | 1.23 |
| NC  +20eq ZnPc |  | 0.46  ±0.01 | 44 | 2.34  ±0.03 | 44 | 8.50  ±0.18 | 12 |  |  |  |  |  | 1.35 |
| NC  +30eq ZnPc |  | 0.40  ±0.01 | 59 | 2.10  ±0.05 | 33 | 7.42  ±0.26 | 8 |  |  |  |  |  | 1.15 |
| NC  +40eq ZnPc |  | 0.33  ±0.01 | 65 | 1.93  ±0.04 | 30 | 7.92  ±0.43 | 5 |  |  |  |  |  | 1.01 |
| NC  +40eq ZnPc | 702 |  |  |  |  |  |  | 0.61  ±0.01 | 3.67  ±0.03 | 79 | 22.27  ±0.8 | 21 | 1.14 |
| ZnPc | 702 |  |  |  |  |  |  |  | 2.77  ±0.03 |  |  |  | 1.14 |

(STX), (X), (DTX), (R), (S_1_), and (S_1_’) indicate shallow trap exciton recombination, direct recombination, deep trap exciton recombination, rise time, singlet excited state, and singlet excited state sensitized from energy transfer.

**Table S2.** Rate and time constants derived from fs-TSA and ns-TAS measurements. Target analysis was applied to obtain respective constants.

| **Sample** | ***k*_hot-X_**  **±sd**  **(ps^-1^)** | ***k*_MX_**  **±sd**  **(ps^-1^)** | ***k*_XX_**  **±sd**  **(ps^-1^)** | ***k*_STX_**  **±sd**  **(ns^-1^)** | ***k*_X_**  **±sd**  **(ns^-1^)** | ***k*_DTX_**  **±sd**  **(ps^-1^)** | ***k*_S1_**  **±sd**  **(ns^-1^)** | ***k*_T1_**  **±sd**  **(μs^-1^)** |
| --- | --- | --- | --- | --- | --- | --- | --- | --- |
| NC | 0.1730  ±0.0022 | 0.0923  ±0.0011 | 0.0296* | 0.99  ±0.05 | 0.228  ±0.003 |  |  |  |
| ZnPc |  |  |  |  |  | 0.0608  ±0.0008 | 0.5230  ±0.0015 | 1.45* |
| NC+40eq ZnPc | 0.1806  ±0.0026 | 0.0847  ±0.0014 | 0.0331  ±0.0006 | 4.08  ±0.06 | 0.42  ±0.01 |  | 0.36  ±0.01 | >0.025* |
| **Sample** | **τ_hot-X_**  **±sd**  **(ps)** | **τ_MX_**  **±sd**  **(ps)** | **τ_XX_**  **±sd**  **(ps)** | **τ_STX_**  **±sd**  **(ns)** | **τ_X_**  **±sd**  **(ns)** | **τ_DTX_**  **±sd**  **(ps)** | **τ_S1_**  **±sd**  **(ns)** | **τ_T1_**  **±sd**  **(μs)** |
| NC | 5.78  ±0.07 | 10.84  ±0.13 | 33.81* | 1.01  ±0.05 | 4.38  ±0.06 |  |  |  |
| ZnPc |  |  |  |  |  | 16.44  ±0.21 | 1.91  ±0.01 | 0.69* |
| NC  +40eq ZnPc | 5.54  ±0.08 | 11.80  ±0.20 | 30.25  ±0.54 | 0.25  ±0.01 | 2.39  ±0.06 |  | 2.75  ±0.07 | >40* |

(hot-X), (MX), (XX), (STX), (X), (DTX), (S_1_), and (T_1_) indicate hot exciton, multiexciton, biexciton, shallow trap exciton recombination, direct exciton recombination, deep trap exciton recombination, singlet excited state, and triplet excited state.

**Table S3.** Rate and time constants from ns-TAS measurements. Target analysis was applied to obtain respective constants.

| **EOS** | ***k*_STX_**  **±sd**  **(ns^-1^)** | ***k*_X_**  **±sd**  **(ns^-1^)** | ***k*_DTX_**  **±sd**  **(ns^-1^)** | ***k*_DT_**  **±sd**  **(μs^-1^)** | ***k*_DT’_**  **±sd**  **(μs^-1^)** | ***k*_S1_**  **±sd**  **(ns^-1^)** | ***k*_T1_**  **±sd**  **(μs^-1^)** |
| --- | --- | --- | --- | --- | --- | --- | --- |
| NC | 1.119  ±0.048 | 0.272  ±0.003 | 0.044  ±0.002 | 7.07* |  |  |  |
| ZnPc |  |  |  |  |  | 0.3820 ±0.0002 | 2.0487 ±0.0005 |
| NC +40eq ZnPc | 3.030* | 0.745  ±0.039 | 0.077  ±0.002 | 10.319 ±0.281 | 0.749  ±0.039 | 0.340  ±0.011 | >0.025 |
| **EOS** | **τ_STX_**  **±sd**  **(ns)** | **τ_X_**  **±sd**  **(ns)** | **τ_DTX_**  **±sd**  **(ns)** | **τ_DT_**  **±sd**  **(μs)** | **τ_DT’_**  **±sd**  **(μs)** | **τ_S1_**  **±sd**  **(ns)** | **τ_T1_**  **±sd**  **(μs)** |
| NC | 0.89  ±0.04 | 3.68  ±0.04 | 22.68  ±1.03 | 0.10* |  |  |  |
| ZnPc |  |  |  |  |  | 2.61  ±0.01 | 0.69  ±0.01 |
| NC +40eq ZnPc | 0.33* | 1.34  ±0.07 | 12.97  ±0.34 | 0.09  ±0.01 | 1.33  ±0.07 | 2.93  ±0.09 | >40 |

(STX), (X), (DTX), (DT), (DT’), (S1) and (T1) indicate shallow trap exciton recombination, direct exciton recombination, deep trap exciton recombination, deep nonemissive trap state, new created deep nonemissive trap state, singlet state and triplet state.
